# Metabolic profiling of CAR T-cells in patients reveals a shift toward amino acid-supported OXPHOS and informs transporter engineering

**DOI:** 10.21203/rs.3.rs-10832865/v1

**Published:** 2026-09-15

**Authors:** Josquin Moraly, Taisuke Kondo, King Chan, Sooraj Achar, Makoto Ando, Marie Pouzolles, Bonnie Yates, Alexandra Dreyzin, Mehdi Benzaoui, Alka Dwivedi, Saliha Majdoul, Justin Mirazee, Jaehyun Suh, Angela Su, Cedric Mongellaz, Hannah Dada, Ye Yang, Swapna Vidhur Daulatabad, Krithika Bhuvaneshwar, Ying Wu, Mina O Seedhom, Valerie Zimmermann, Sandrina Kinet, Daniel Crooks, Hannah Song, Jonathan W Yewdell, Christopher Chien, Olivier Hermine, Valerie Dardalhon, Steven L. Highfill, Thorkell Andresson, Grégoire Altan-Bonnet, Nirali N. Shah, Naomi Taylor

**Affiliations:** 1 Pediatric Oncology Branch, Center for Cancer Research, National Cancer Institute, National Institutes of Health, Bethesda, MD, USA; 2 Laboratory of Integrative Cancer Immunology, Center for Cancer Research, National Cancer Institute, National Institutes of Health, Bethesda, MD, USA; 3 Protein Characterization Laboratory, Frederick National Laboratory for Cancer Research National Institutes of Health, Frederick, MD, USA; 4 Université de Montpellier, Institut de Génétique Moléculaire de Montpellier, Montpellier, France; 5 Cellular Biology Section, Laboratory of Viral Diseases, National Institute of Allergy and Infectious Diseases, National Institutes of Health, Bethesda, MD 20892, USA; 6 Center for Cellular Engineering, Department of Transfusion Medicine, National Institutes of Health, Bethesda, MD, USA; 7 Urologic Oncology Branch, Center for Cancer Research, National Cancer Institute, National Institutes of Health, Bethesda, MD, USA; 8 Institut des Maladies Génétiques (IMAGINE), Université de Paris, INSERM, Paris, France

## Abstract

CAR T-cell efficacy requires post-infusion expansion and persistence, yet metabolic programs supporting T-cells in patients remain poorly defined. Here, we developed a high-throughput single-cell immunometabolic profiling pipeline and applied it across pediatric leukemia trials, revealing a conserved post-infusion CAR T-cell shift from glycolysis toward amino acid-driven oxidative phosphorylation (OXPHOS). Within this remodeling, OXPHOS-dependent stem-like CAR T-cell subsets were enriched in patients achieving complete remission. Longitudinal plasma metabolomics revealed cytokine release syndrome-associated depletion of multiple amino acids, including glutamine and arginine, during CAR T-cell expansion, creating a nutrient-restricted environment. Analysis of public CAR T-cell datasets showed that responders upregulated amino-acid solute carrier transporters, whereas disrupting uptake impaired translation, OXPHOS, stemness, and cytotoxicity. Guided by these findings, we screened amino-acid transporters for CAR T-cell engineering. SLC1A5-, SLC7A1-, and SLC38A9-armored CAR T-cells emerged as the most promising, with enhanced oxidative capacity and anti-leukemic efficacy, establishing amino acid transport as a targetable metabolic checkpoint.

## INTRODUCTION

Chimeric antigen receptor (CAR) T-cell therapies have transformed the treatment of B-cell malignancies, yet durable remission remains restricted to a subset of patients^1–3^. Across clinical trials, the magnitude and durability of CAR T-cell expansion following infusion are among the most consistent correlates of therapeutic response, underscoring cellular fitness after adoptive transfer as a key determinant of clinical outcome^2–4^. From a bioenergetic perspective, sustained antitumor T-cell responses require extensive metabolic support to fuel proliferation, migration, and effector function, as well as to regulate signaling, transcriptional programs, and epigenetic states^5–7^. Accordingly, cell-intrinsic metabolism has emerged as a critical determinant of T-cell persistence and antitumor activity^8–11^. However, the specific metabolic programs that support CAR T-cell function, particularly after infusion into patients, remain poorly defined.

Importantly, CAR T-cell metabolism is not merely descriptive but functionally actionable. A growing body of preclinical work has demonstrated that manipulation of metabolic enzymes, nutrient transporters, and regulatory pathways—including arginine, glutamine, and glucose metabolism—can enhance CAR T-cell persistence and antitumor efficacy across hematologic and solid tumor models. These studies highlight the therapeutic potential of metabolic rewiring^5,12,13^. At the same time, metabolic interventions have often yielded context-dependent and sometimes divergent outcomes, reflecting differences in tumor type, microenvironmental constraints, and experimental systems^14^. Moreover, most approaches have been evaluated *in vitro* or in murine models^14–16^ frequently under conditions that fail to recapitulate the metabolic pressures encountered by CAR T-cells after infusion into patients. Thus, despite compelling evidence that metabolism can be leveraged to improve CAR T-cell performance, the metabolic wiring that sustains durable CAR T-cell responses *in vivo* remains unclear.

Preclinical studies have further shown that costimulatory signaling via CD28 or 4-1BB shapes T-cell metabolic fate by promoting coordinated metabolic remodeling and mitochondrial biogenesis, with 4-1BB signaling generally imparting a stronger bias toward oxidative metabolism that favors memory-like states^17–19^. CARs incorporating these domains similarly exhibit distinct metabolic and phenotypic features *in vitro* and following infusion into mice or patients^20,21^. However, clinical experience increasingly challenges a simple costimulation-based framework: CAR constructs with similar costimulatory architectures can display divergent persistence, efficacy, and toxicity profiles^22–25^ and recent patient-based analyses suggest that CAR T-cells from responders may converge on shared metabolic features despite distinct CAR designs^21,26^. Together, these findings suggest that CAR T-cell metabolic fitness *in vivo* is shaped by factors beyond costimulatory architecture alone.

An additional and largely unexplored challenge is the acute metabolic shift to which CAR T-cells are exposed during therapy. CAR T-cell products are generated under nutrient-rich, hyperoxic *ex vivo* conditions and then abruptly transferred into chemotherapy-pretreated patients characterized by acute leukemia, baseline lymphodepletion, and systemic inflammation. Severe inflammatory states such as cytokine release syndrome (CRS) are accompanied by profound alterations in systemic metabolism that may restrict nutrient availability^27–30^. How CAR T-cells adapt metabolically to this drastic environmental transition, and which metabolic states support sustained expansion, persistence, and antitumor function in patients, remain unclear. Furthermore, while oxidative phosphorylation (OXPHOS) and amino acid metabolism play central roles in sustaining T-cell function in physiologic conditions in mice^31–34^, it is not known whether similar programs are engaged by CAR T-cells in patients, and whether nutrient uptake pathways can be exploited to overcome systemic nutrient stress.

Here, we apply a functional single-cell translation and metabolic profiling approach to pediatric and adolescent and young adult (AYA) patients with B-cell acute lymphoblastic leukemia (B-ALL) to demonstrate that CAR T-cells undergo a profound metabolic transition after infusion. While *ex vivo* products are predominantly glycolytic, post-infusion CD22 and CD19/CD22 CAR T-cells shift toward an oxidative program marked by increased dependence on amino acid–driven OXPHOS. Within this transition, this single-cell profiling framework identifies discrete CAR T-cell subsets distinguished by their reliance on OXPHOS-coupled protein translation, which strongly correlates with enhanced expansion, stem-like memory differentiation, and clinical response. We further demonstrate through longitudinal plasma metabolomics that clinical CRS in our pediatric/AYA cohort drives a severe systemic depletion of multiple circulating amino acids—most prominently glutamine and arginine—as well as associated tryptophan derivatives, creating a nutrient-stressed *in vivo* environment. Guided by re-analysis of publicly available clinical single-cell transcriptomic datasets^35^, we identified amino acid transport as a candidate metabolic vulnerability and engineered CAR T-cells to overexpress selected amino acid solute carrier (SLC) transporters. SLC1A5 (ASCT2), SLC7A1 (CAT1), and SLC38A9 each enhanced mitochondrial metabolism and improved antitumor activity under stringent metabolic stress conditions *in vitro* and *in vivo*. Together, these findings establish amino acid–supported OXPHOS as a central determinant of CAR T-cell performance and identify metabolic armoring via SLC transporters as a rational strategy to enhance CAR T-cell fitness in nutrient-restricted environments.

## RESULTS

### High-throughput immuno-metabolic profiling resolves CAR construct-specific states and identifies protein synthesis as an integrative single-cell readout.

**Figure 1.**
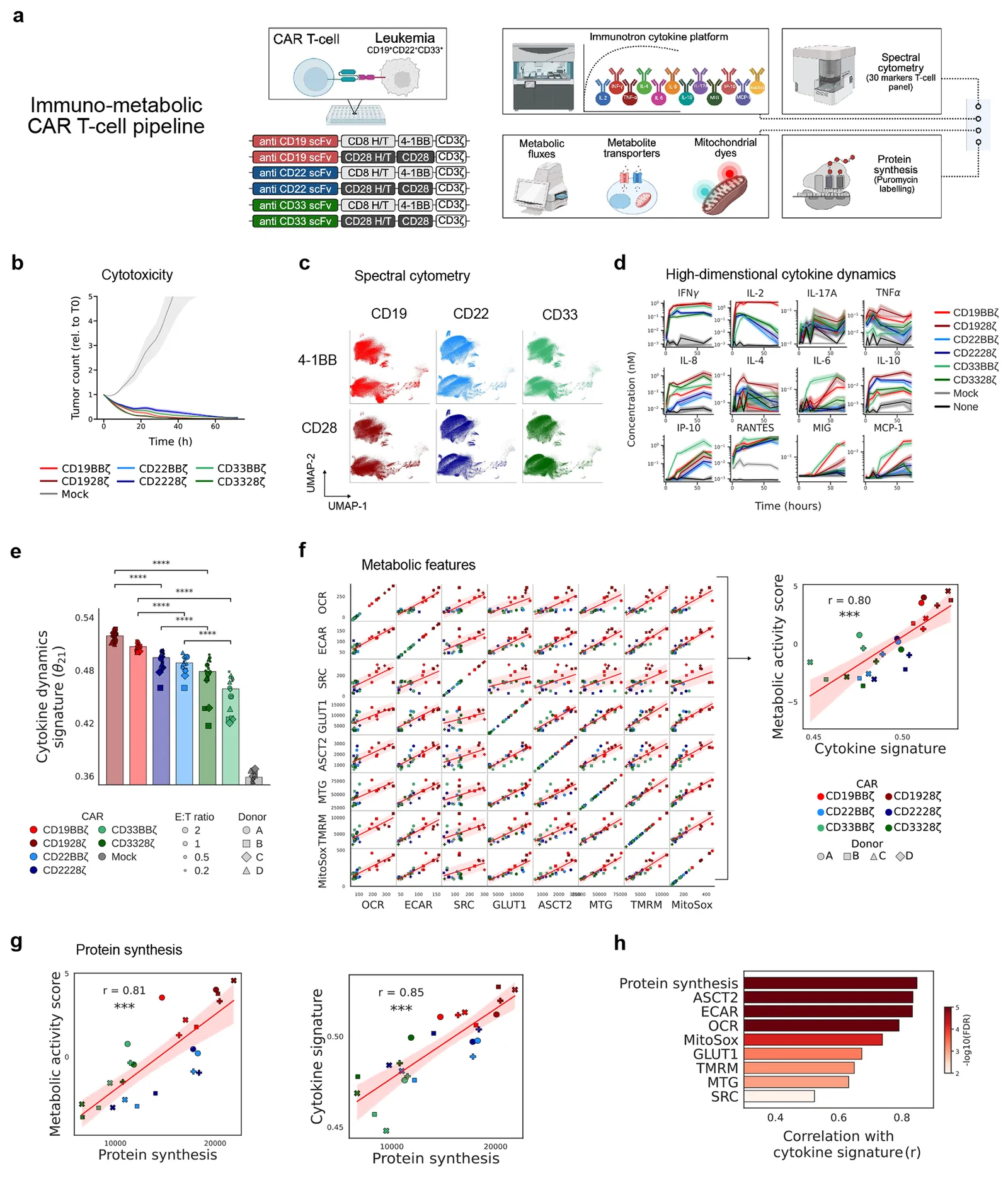
Protein synthesis captures integrated metabolic and functional CAR T-cell states. **a,** Overview of the immuno-metabolic CAR T-cell pipeline. Primary human T-cells from four donors were transduced with CD19, CD22, or CD33 CARs containing either CD28 or 4-1BB costimulatory domains and stimulated with NALM6 leukemia cells expressing CD19, CD22, and CD33 antigens. Cytotoxicity, cytokine secretion dynamics, metabolic parameters, protein synthesis, and spectral cytometry measurements were acquired following activation. **b,** Incucyte cytotoxicity assay using NALM6 target cells at an effector-to-target (E:T) ratio of 1:1 across all CAR constructs. One representative donor is shown. **c,** UMAP representation of spectral cytometry data from CAR T-cells 72 h after activation across all CAR constructs, donors, and effector-to-target (E:T) ratios (2:1, 1:1, 0.5:1, and 0.2:1). **d,** High-throughput cytokine and chemokine secretion profiling using the *IMMUNOtron* platform. Secretion of 12 cytokines and chemokines was measured at 12 time points over 72 h. One representative donor is shown (E:T ratio 1:1). **e,** Cytokine dynamics signatures across CAR constructs. The cytokine dynamics signature (θ21), an integrated measure of cytokine secretion dynamics over time, is shown for each CAR construct across donors. **f,** Coordinated metabolic remodeling across CAR constructs. Individual metabolic parameters—including oxygen consumption rate (OCR), extracellular acidification rate (ECAR), spare respiratory capacity (SRC), surface expression of the glucose transporter SLC2A1 (GLUT1) and glutamine transporter SLC1A5 (ASCT2), mitochondrial mass (MTG), mitochondrial membrane potential (TMRM), and mitochondrial reactive oxygen species (MitoSOX)—were quantified and their crosscorrelations are shown (left). Metabolic features were integrated into a weighted PCA-derived composite metabolic activity score, and its correlation with the cytokine dynamics signature (θ21) is shown (right). **g,** Correlations between protein synthesis, the cytokine dynamics signature (θ21), and the composite metabolic activity score across CAR constructs and donors. **h,** Relationships between cytokine secretion dynamics (θ_21_) and individual metabolic features. Statistical significance was determined using two-tailed paired Student’s *t*-tests (e) or Pearson correlation analysis with Benjamini–Hochberg correction for multiple comparisons (f, g, h). *P* values are indicated as follows: *P < 0.05, **P < 0.01, ***P < 0.001, and ****P < 0.0001; ns, not significant.

Although CAR constructs often exhibit comparable cytotoxic activity in vitro, their clinical efficacy can differ substantially^23,24,36^, highlighting the need for quantitative functional metrics capable of distinguishing construct-specific states. High-dimensional cytokine and metabolic profiling offer approaches to characterize T-cell functional states beyond conventional cytotoxicity and phenotypic assays^37,38^, but whether these approaches can systematically resolve CAR construct-specific states remains unclear. To address this challenge, we developed an integrated immuno-metabolic profiling framework to compare functional, phenotypic, and metabolic states across CAR constructs (Fig. 1a). Using this approach, we benchmarked six CAR constructs targeting CD19, CD22, or CD33 and incorporating either CD28 or 4-1BB costimulatory domains.

All CAR constructs were efficiently transduced, exhibited comparable CD4/CD8 ratios (**Extended Data Fig. 1a**), and effectively killed antigen-expressing tumor cells (Fig. 1b), making them difficult to distinguish using conventional cytotoxicity assays. Likewise, UMAP projection of 30 cell-surface markers failed to clearly separate individual CAR constructs (Fig. 1c). High-throughput cytokine and chemokine secretion profiling over 72 hours using our robotic *IMMUNOtron* platform^37^ clearly distinguished CAR constructs across donors and effector-to-target ratios (Fig. 1d **and Extended Data Fig. 1c**), but total secretion alone did not establish a clear functional hierarchy. We therefore projected time-resolved cytokine responses into low-dimensional space revealing construct-specific response trajectories (**Extended Data Fig. 1d**). The angular trajectory parameter (θ_21_) distilled these trajectories into a composite cytokine signature that robustly resolved construct-specific functional states and revealed CAR-dependent differences in the impact of CD28 versus 4-1BB costimulation (Fig. 1e and **Extended Data Fig. 1e**).

We next asked whether these cytokine signatures reflected differences in metabolic states. Metabolic parameters were highly correlated across conditions, indicating coordinated regulation of nutrient uptake, mitochondrial function, and bioenergetic activity following CAR activation (Fig. 1f). Oxygen consumption positively correlated with glycolytic activity, demonstrating that highly functional CAR T-cells simultaneously engage oxidative and glycolytic metabolism rather than adopting mutually exclusive metabolic states (Fig. 1f). Increased metabolic activity was accompanied by coordinated increases in nutrient transporter expression, mitochondrial mass, and mitochondrial activity (Fig. 1f). Integration of these parameters into a composite metabolic activity score revealed a strong correlation with the cytokine signature and with specific cell-surface markers that identified CAR T-cell clusters with high or low metabolic activity (Fig. 1f **and Extended Data Fig. 1f–h**). Together, these findings demonstrate that construct-specific functional programs are tightly linked to coordinated metabolic remodeling.

A remaining challenge, however, was identifying a single quantitative metric applicable to clinical samples, where post-infusion cell numbers are limiting. As the largest ATP-consuming cellular process, protein synthesis depends on coordinated nutrient uptake, mitochondrial metabolism, and cellular bioenergetics^39–41^, making it an attractive candidate for integrating these programs. Protein synthesis, quantified by puromycin incorporation, increased linearly over time in both primary T cells and ex vivo-expanded CAR T-cells, providing a robust quantitative readout (**Extended Data Fig. 2a–b**). Consistent with its central role in coupling nutrient availability and bioenergetic capacity to cellular function^42–46^, protein synthesis strongly correlated with the composite metabolic activity score and the cytokine signature (Fig. 1g). Moreover, among all metabolic parameters evaluated, protein synthesis exhibited the strongest association with the cytokine signature and, more broadly, with individual cytokines and activation markers (Fig. 1h **and Extended Data Fig. 1f**). Together, these findings identify protein synthesis as a quantitative single-cell metric that captures integrated metabolic and functional states across CAR constructs and is readily applicable to the metabolic profiling of limited clinical CAR T-cell samples, enabling direct interrogation of post-infusion CAR T-cell metabolism in patients.

### CAR T-cells undergo a post-infusion metabolic shift from glycolysis to glutamine-supported OXPHOS

**Figure 2.**
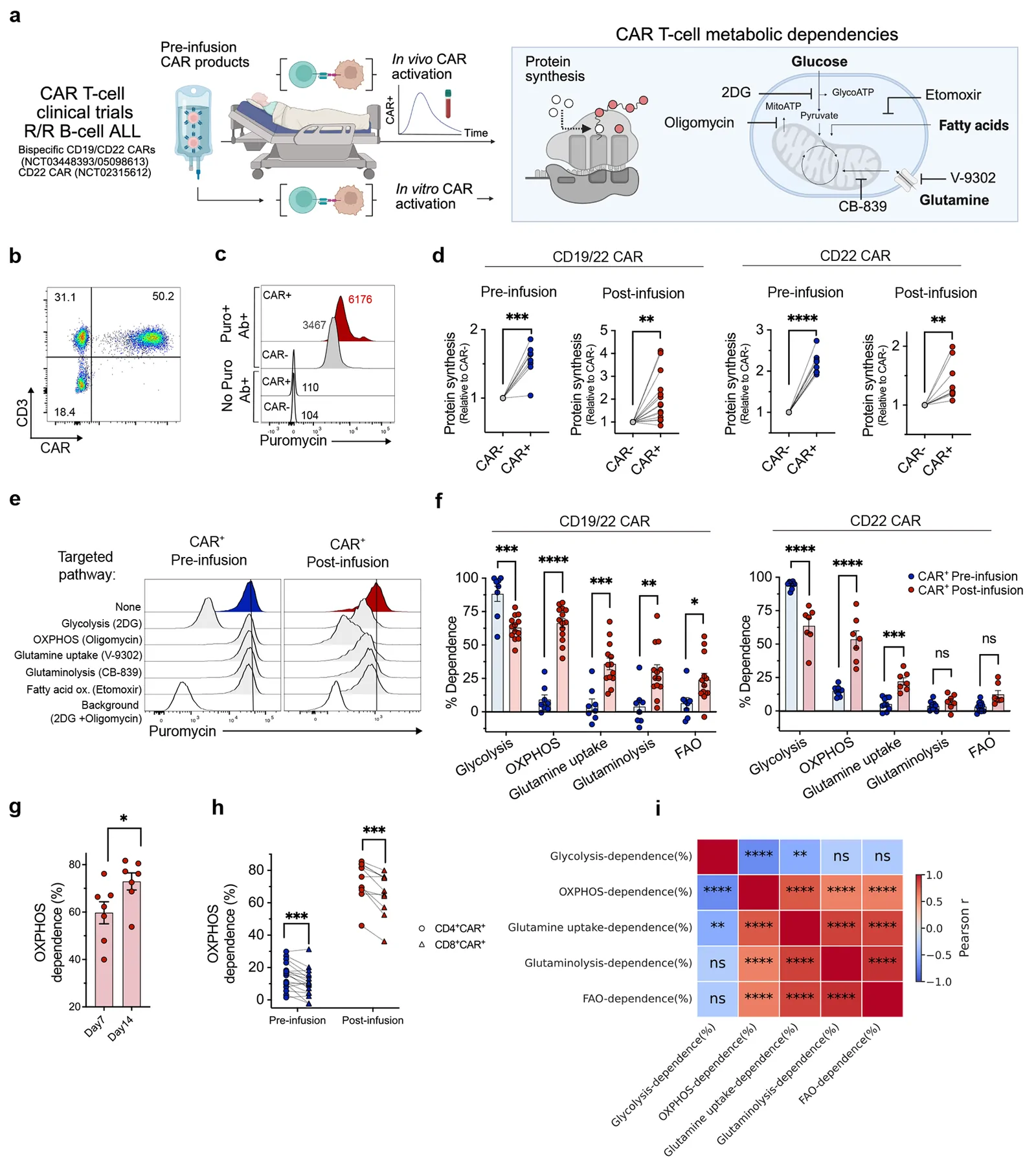
CAR T-cells undergo a post-infusion metabolic shift from glycolysis to glutamine-supported OXPHOS. **a,** Study design. Infusion products and post-infusion peripheral blood samples from anti-CD22 (NCT02315612) and bispecific anti-CD19/CD22 (NCT03448393, NCT05098613) CAR T-cell trials were analyzed using puromycin incorporation following inhibition of glycolysis (2DG), OXPHOS (Oligomycin), glutamine uptake (V-9302), glutaminolysis (CB-839), or fatty acid oxidation (Etomoxir). Infusion products were activated by co-culture with NALM6 cells for 72 h. **b–c**, Protein synthesis in circulating CAR^+^ and CAR^−^ T-cells following infusion. Representative flow cytometry plots and quantification are shown. **d,** Relative protein synthesis in CAR^+^ and CAR^−^ T-cells from pre-infusion and post-infusion samples. **e,** Metabolic dependencies supporting protein synthesis in pre-infusion (blue) and post-infusion (red) CAR T-cells from a representative patient. **f,** Metabolic dependencies supporting protein synthesis in infusion products (blue) and post-infusion (red) CAR T-cells from CD22 and CD19/CD22 CAR T-cell trials. **g,** OXPHOS dependence in CAR T-cells at days 7 and 14 following infusion. **h,** Paired comparison of OXPHOS dependence between CAR^+^CD4^+^ and CAR^+^CD8^+^ T cells within individual samples. **i,** Correlation analysis of metabolic dependencies across infusion products and post-infusion CAR T-cells from both clinical trials. Statistical comparisons were performed using two-tailed paired Student’s *t*-tests (d, h), two-tailed unpaired Student’s *t*-tests (f–g), and Pearson correlation analysis with Benjamini–Hochberg false discovery rate correction (i). *P* values are indicated as follows: *P < 0.05, **P < 0.01, ***P < 0.001, and ****P < 0.0001; ns, not significant.

Having established protein synthesis as a scalable single-cell readout of CAR T-cell metabolic state (Fig. 1), we next interrogated metabolic dependencies in CAR T-cells from pediatric and AYA patients with R/R B-cell B-ALL treated with either CD22 CAR T-cell therapy (NCT02315612; *n*=22 samples, 12 patients) or bispecific CD19/CD22 CAR T-cell therapy (NCT03448393/NCT05098613; *n*=28 samples, 9 patients) (Fig. 2a; **Extended Data Tables 1 and 2)**. Protein synthesis was quantified in pre-infusion CAR T-cell products and post-infusion peripheral blood samples collected during the expansion phase (days 7–14) under baseline conditions and following inhibition of major metabolic pathways (Fig. 2a). Following infusion, circulating CAR^+^ T-cells identified by CAR staining (Fig. 2b) exhibited significantly higher protein synthesis than CAR^−^ T-cells across both trials (*p*<0.01, Fig. 2c–2d), consistent with *in vivo* activation.

To define how CAR T-cell metabolism changes following infusion, we quantified the contribution of glycolysis, OXPHOS, glutamine uptake, glutaminolysis, and fatty acid oxidation (FAO) to protein synthesis using selective metabolic inhibitors (Fig. 2e **and Extended Data Fig. 2c)**, with metabolic dependence inferred from changes in protein translation (Fig. 2e). Whereas both CD22 and CD19/22 CAR T-cell infusion products were overwhelmingly dependent on glycolysis (mean dependencies of 94.6% and 88.3%, respectively), they exhibited limited reliance on OXPHOS, glutaminolysis, or FAO (<15%, Fig. 2f). In contrast, post-infusion CAR T-cells from all three trials underwent a striking metabolic transition toward oxidative metabolism. OXPHOS dependence increased from 15.1% to 53.5% in CD22 CAR T-cells and from 9.2% to 66.4% in CD19/CD22 CAR T-cells (*p*<0.0001, Fig. 2f). This transition was fueled by a marked increase in glutamine metabolism, with dependence on glutamine uptake increasing 4-7-fold following infusion (*p*<0.0001, Fig. 2f). Increased reliance on glutaminolysis and FAO was also observed, particularly in CD19/CD22 CAR T-cells. Moreover, this oxidative program continued to intensify between day 7 and day 14 following infusion (*p* = 0.04, Fig. 2g).

Subset analysis revealed consistently greater OXPHOS reliance in CD4^+^ compared with CD8^+^ CAR^+^ T-cells, both before and after infusion (*p*<0.001, Fig. 2h). Across all samples, glycolytic dependence inversely correlated with mitochondrial dependence, whereas OXPHOS dependence positively correlated with glutamine uptake, glutaminolysis, and FAO (*p* = 0.01-0.0001, Fig. 2i). Together, these findings demonstrate profound metabolic remodeling of CAR T-cells following infusion, characterized by a transition from glycolysis-dominated metabolism toward an amino acid-supported oxidative program.

### OXPHOS-dependent CAR T-cell subsets in infusion products and post-infusion samples predict expansion and complete responses

**Figure 3.**
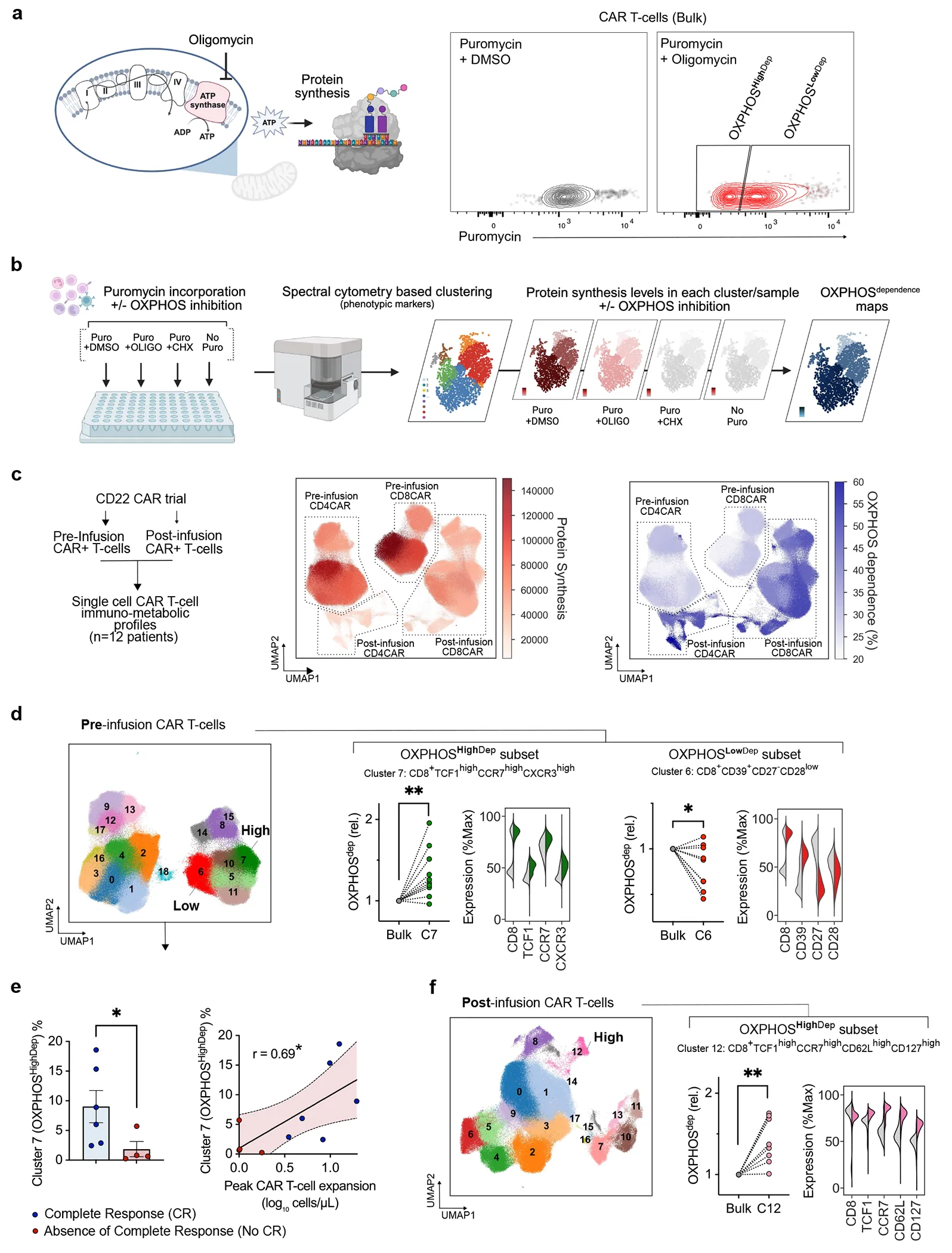
Single-cell metabolic profiling identifies OXPHOS-dependent CAR T-cell subsets associated with expansion and clinical response. **a,** Schematic illustrating inhibition of ATP synthase by oligomycin and its impact on protein synthesis measured by puromycin incorporation (left). Representative flow plots of CAR T cells from a patient following ATP synthase inhibition demonstrate heterogeneity in protein synthesis, distinguishing cells with low (OXPHOS^LowDep^) and high (OXPHOS^HighDep^) dependence on OXPHOS (right). **b,** Schematic of the single-cell metabolic profiling assay combining a 29-marker spectral flow cytometry panel with assessment of OXPHOS dependence through oligomycin-mediated inhibition of protein synthesis. **c,** Single-cell metabolic profiling of pre-infusion CAR T-cell products and post-infusion PBMCs collected during the expansion phase of the CD22 CAR T-cell trial. UMAPs of CD4^+^ and CD8^+^ CAR T-cell PARC clusters are shown and colored according to protein synthesis (red) or OXPHOS dependence (blue). **d,** Identification of pre-infusion CAR T-cell subsets with distinct levels of OXPHOS dependence. Clusters exhibiting increased (High) or decreased (Low) OXPHOS dependence relative to the bulk CAR T-cell population are indicated (left). Relative OXPHOS dependence of individual patient samples compared with the bulk population (grey), and phenotypic characterization of the OXPHOS-high cluster C7 (OXPHOS^HighDep^, middle) and OXPHOS-low cluster C6 (OXPHOS^LowDep^, right). Phenotypes are shown as half-violin plots, with the bulk population shown in grey and cluster-specific profiles shown in color. **e,** Frequency of OXPHOS-high Cluster C7 in patients achieving complete remission (CR, blue) or not achieving complete remission (No CR, red) (left). Association between Cluster C7 frequency and *in vivo* CAR T-cell expansion (right). **f,** Identification of a post-infusion CAR T-cell subset with high levels of OXPHOS dependence. The OXPHOS-high cluster C12 is indicated (left). Relative OXPHOS dependence of individual patient samples compared with the bulk population (gray) and phenotypic characterization of cluster C12 (OXPHOS^HighDep^, right). Phenotypes are shown as half-violin plots, with the bulk population shown in gray and cluster-specific profiles shown in color. Statistical significance was determined using Wilcoxon signed-rank tests (d, f), Pearson correlation analysis (e, right), and Welch’s two-sided unpaired *t*-tests (e). *P* values are indicated as follows: *P < 0.05, **P < 0.01, ***P < 0.001, and ****P < 0.0001; ns, not significant.

The analyses above revealed a global shift toward OXPHOS following CAR T-cell infusion but provided limited insight into the metabolic states of individual T-cell subsets. Notably, inhibition of ATP synthase identified functionally distinct populations: cells that maintained protein synthesis despite OXPHOS inhibition (OXPHOS^LowDep^) and cells exhibiting marked reductions in protein synthesis (OXPHOS^HighDep^; Fig. 3a). To further characterize this metabolic diversity, we developed a single-cell metabolic profiling assay that combines oligomycin-based assessment of OXPHOS dependence with a 29-marker spectral flow cytometry panel (Fig. 3b **and Extended Data Fig. 3a)**. OXPHOS dependence was then mapped across phenotypically defined cell clusters based on oligomycin-induced changes in protein synthesis. Assay performance and reproducibility were first validated in peripheral blood T-cells from healthy donors (**Extended Data Fig. 3b–e**), revealing marked differences in protein synthesis and OXPHOS dependence across naïve, memory, effector, and regulatory T-cell subsets (**Extended Data Fig. 3f–g**). These metabolic profiles were highly reproducible across donors (**Extended Data Fig. 3h-i**).

We next applied this approach to CD22 CAR T-cell products and matched post-infusion samples from 12 patients (21 samples total; Fig. 3c), profiling approximately 1.4×10^5^ CD3^+^CAR^+^ cells, including 8.1×10^4^ from infusion products and 6.0×10^4^ from post-infusion samples. Unsupervised clustering revealed clear segregation between pre- and post-infusion CAR T-cell states in both CD4^+^ and CD8^+^ subsets. Protein synthesis was highest in recently activated infusion products (Fig. 3c), whereas OXPHOS dependence was markedly enriched in post-infusion CAR T-cell populations (Fig. 3c), consistent with the metabolic remodeling identified in bulk analyses (Fig. 2). Among 18 pre-infusion CAR^+^ clusters identified (Fig. 3d, **Extended Data Fig. 4a**), a CD8^+^TCF1^High^CCR7^High^CXCR3^High^ subset (Cluster C7) exhibited significantly higher OXPHOS dependence than the bulk CAR T-cell population. Importantly, the frequency of this OXPHOS^HighDep^ subset strongly correlated with *in vivo* expansion (r=0.69; Fig. 3e) and was significantly enriched in patients achieving complete remission (CR) compared with non-responders (NR, Fig. 3e). In contrast, a CD8^+^CD39^High^ subset (Cluster C6), previously associated with suboptimal responses to adoptive T-cell therapy^47^, exhibited significantly lower OXPHOS dependence than the population mean (Fig. 3d, **Extended Data Fig. 4a**).

Analysis of post-infusion CAR T-cell populations identified an additional OXPHOS^HighDep^ subset of interest (Fig. 3f, **Extended Data Fig. 4b**). Cluster C12 exhibited a stem-like phenotype characterized by expression of TCF1, CCR7, CD62L, and CD127 and displayed significantly elevated OXPHOS dependence relative to the bulk population, consistent with a self-renewal state (Fig. 3f). In contrast, a terminally differentiated CD8^+^CD28^−^CD27^−^TCF1^low^ subset (Cluster C8), analogous to the TCF1^−^ exhausted populations previously associated with resistance to checkpoint blockade^48,49^, was significantly enriched in NR patients (**Extended Data Fig. 4c**). Together, these findings identify discrete OXPHOS^HighDep^ CAR T-cell populations associated with stem-like phenotypes, enhanced *in vivo* expansion, and complete clinical responses, establishing metabolic state as a key determinant of CAR T-cell persistence and therapeutic efficacy in patients with relapsed/refractory leukemia.

### CAR T-cell expansion and cytokine release syndrome generate an amino acid-depleted environment in patients

**Figure 4.**
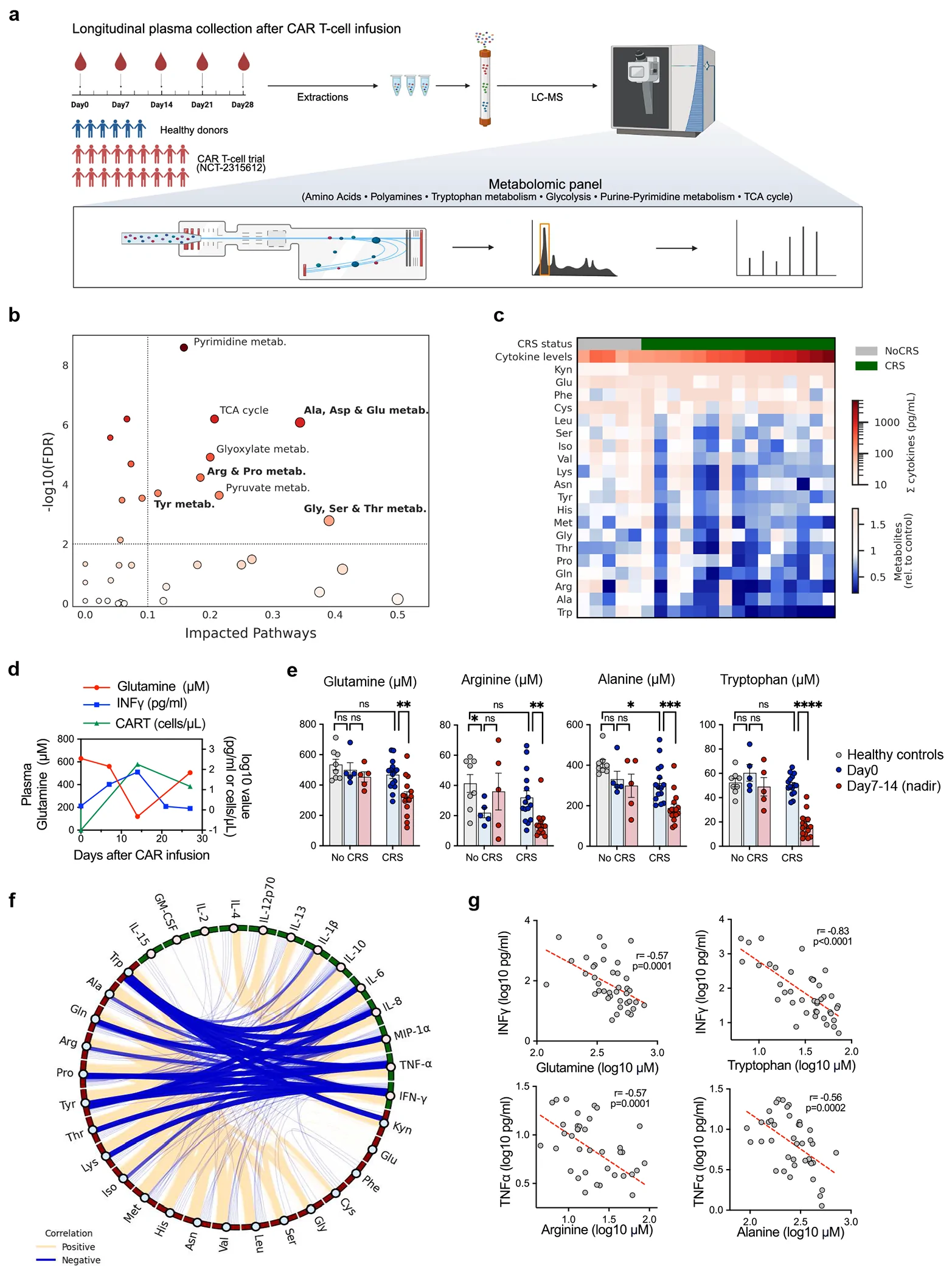
Systemic cytokine release following CAR T-cell infusion drives significant reduction of specific plasma amino acids. **a,** Study design: plasma samples were collected following lymphodepletion and at weekly intervals for 28 days after CAR T-cell infusion. A targeted metabolomics platform was used for absolute quantification of host- and microbiota-derived metabolites. **b,** Metabolic pathways altered in patients during CAR T-cell expansion, based on plasma metabolite levels measured at days 7 and 14, compared with healthy donors. **c,** Changes in plasma amino acid levels in all patients at day 14, color-coded by fold change relative to the median of healthy controls. Samples are ordered by cytokine release syndrome occurrence and circulating cytokine levels. **d,** Plasma levels of glutamine, interferon-γ (IFN-γ), and circulating CAR T-cells in peripheral blood measured over time in a representative patient who exhibited robust CAR T-cell expansion and developed cytokine release syndrome (CRS). **e,** Plasma levels of glutamine, arginine, alanine, and tryptophan compared between healthy controls (grey) and CAR T-cell–treated patients with or without cytokine release syndrome (CRS) at day 0 (blue) and at the nadir between days 7–14 after infusion (red). **f,** Circular ribbon plot detailing global Spearman correlations between circulating amino acids and cytokine levels at the time of CAR T-cell expansion (days 7 and 14). Bands indicate robust, uniform negative correlations between multiple amino acids and inflammatory cytokines, alongside positive intra-amino acid clustering. **g,** Representative Spearman correlation scatter plots demonstrating linear, inverse distributions (slashed lines) between specific cytokine drivers and key amino acid pools (IFN-γ vs. glutamine, IFN-γ vs. tryptophan, TNF-α vs. arginine, and TNF-α vs. alanine) during CAR T-cell expansion. Statistical significance was determined using two-tailed unpaired Student’s *t*-tests (e, healthy controls vs. day 0), two-tailed paired *t*-tests (e, day 0 vs. day 14), and Spearman correlation analysis (f–g). *P* values are indicated as follows: *P < 0.05, **P < 0.01, ***P < 0.001, and ****P < 0.0001; ns, not significant.

The association of OXPHOS-dependent CAR T-cell subsets with complete response, together with the post-infusion shift toward OXPHOS and increased dependence on glutamine utilization, suggested a central role for amino acid metabolism in sustaining CAR T-cell bioenergetics *in vivo*. Because activated T-cells rely on extracellular amino acids to support biosynthesis and mitochondrial metabolism^50–52,13,53^, changes in amino acid availability could directly influence CAR T-cell metabolic state and persistence. However, changes in circulating amino acid pools following CAR T-cell therapy remains poorly characterized. We hypothesized that the plasma metabolome may be shaped by multiple interacting factors, including underlying leukemia, pre-infusion lymphodepletion, and inflammation-associated toxicities such as cytokine release syndrome (CRS), consistent with observations in other inflammatory settings, including graft-versus-host disease (GVHD) and COVID-19^27,28^. To test this hypothesis, we performed longitudinal plasma metabolomic profiling in our pediatric and young adult patients with B-ALL treated with CD22 CAR T-cells, alongside age-matched healthy controls (*n*=88 samples; 20 patients; n=8 healthy donors). A custom targeted metabolomic panel enabled absolute quantification of metabolites spanning key metabolic pathways and classes, including glycolysis, amino acids, tryptophan and indole derivatives, polyamines, the TCA cycle, purine and pyrimidine metabolism, short-chain fatty acids (Fig. 4a).

Profound alterations in systemic metabolism were observed after CAR T-cell infusion relative to healthy controls. During the CAR T-cell expansion phase, four of the eight most significantly affected metabolic pathways were directly amino acid–related (Fig. 4b), underscoring a broad disruption of systemic amino acid metabolism during CAR T-cell responses. Whereas metabolites associated with the TCA cycle, pyrimidine metabolism, and pyruvate metabolism showed abnormalities at baseline and post-infusion time points, amino acid perturbations were largely confined to the period of CAR T-cell expansion, suggesting that these latter changes were not driven by baseline disease or lymphodepletion chemotherapy **(Extended Data Fig. 5a)**.

At the peak of CAR T-cell expansion (14 ± 4.1 days, SD), plasma concentrations of multiple amino acids—most prominently glutamine, arginine, alanine, and tryptophan—were markedly reduced compared with healthy controls, specifically in patients who developed CRS (Fig. 4c). Strikingly, these amino acid levels declined sharply between days +7 and +14 in patients with CRS, yet remained largely stable in non-CRS patients (Fig. 4d–e, **Extended Data 5a–b)**. Moreover, the decrease in tryptophan was accompanied by corresponding decreases in microbiota-derived tryptophan metabolites, including indole-3-carboxaldehyde (ICA), 3-indoxyl sulfate (3-IS), and 3-indole acetic acid (3-IAA) (**Extended Data Fig. 5c**). While the pathophysiological significance of these metabolites in patients receiving CAR T-cell therapy remains unclear, microbiota-derived indole metabolites are increasingly recognized as regulators of immune responses in infection, graft-versus-host disease, and cancer^27,54,55^. Consistent with the potential immunoregulatory role of these metabolites, recent studies in B-cell lymphoma have linked *Akkermansia muciniphila*-associated indole metabolites with improved clinical responses to CD19 CAR T-cell therapy^56^.

To further evaluate the link between amino acid depletion and systemic inflammation, we quantified circulating cytokines and chemokines (IL-1β, IL-2, IL-4, IL-6, IL-8, IL-10, IL-13, IL-15, IL-12, IFN-γ, TNF-α, GM-CSF, MIP-1α) in parallel with plasma metabolite levels at day +14 post-infusion. Mapping these interactions globally using a circular ribbon plot revealed a tight biological coupling and complete cross-concordance between systemic amino acid depletion and inflammatory cytokine surges (Fig. 4f). Across this network, a broad panel of individual amino acids—including tyrosine, alanine, glutamine, arginine, proline, tryptophan, threonine, lysine, isoleucine, and methionine—correlated strongly and negatively with pro-inflammatory and regulatory cytokines (IL- 1β, IL-6, IL-8, IL-10, MIP-1α, TNF-α), while displaying robust positive correlations with each other. Strikingly, kynurenine emerged as the singular metabolic exception, correlating positively with inflammatory markers, consistent with accelerated tryptophan catabolism via the indoleamine 2,3-dioxygenase (IDO) pathway. This absolute coordination is directly highlighted by representative pairwise correlation scatter plots, which display strict, negative linear distributions between key inflammatory drivers and primary nutrient pools, specifically IFN-γ/glutamine, IFN-γ/tryptophan, TNF-α/arginine, and TNF-α/alanine (Fig. 4g). Together, these findings demonstrate that CAR T-cell expansion and its associated CRS radically reshape the systemic metabolic landscape, generating an amino acid–depleted, nutrient-stressed environment. This profound metabolic remodeling likely constrains CAR T-cell effector function, particularly in the context of the adaptive shift toward amino acid–supported OXPHOS observed in post-infusion CAR T-cells.

### SLC-mediated amino acid uptake supports CAR T-cell OXPHOS, stemness, and cytotoxicity

**Figure 5.**
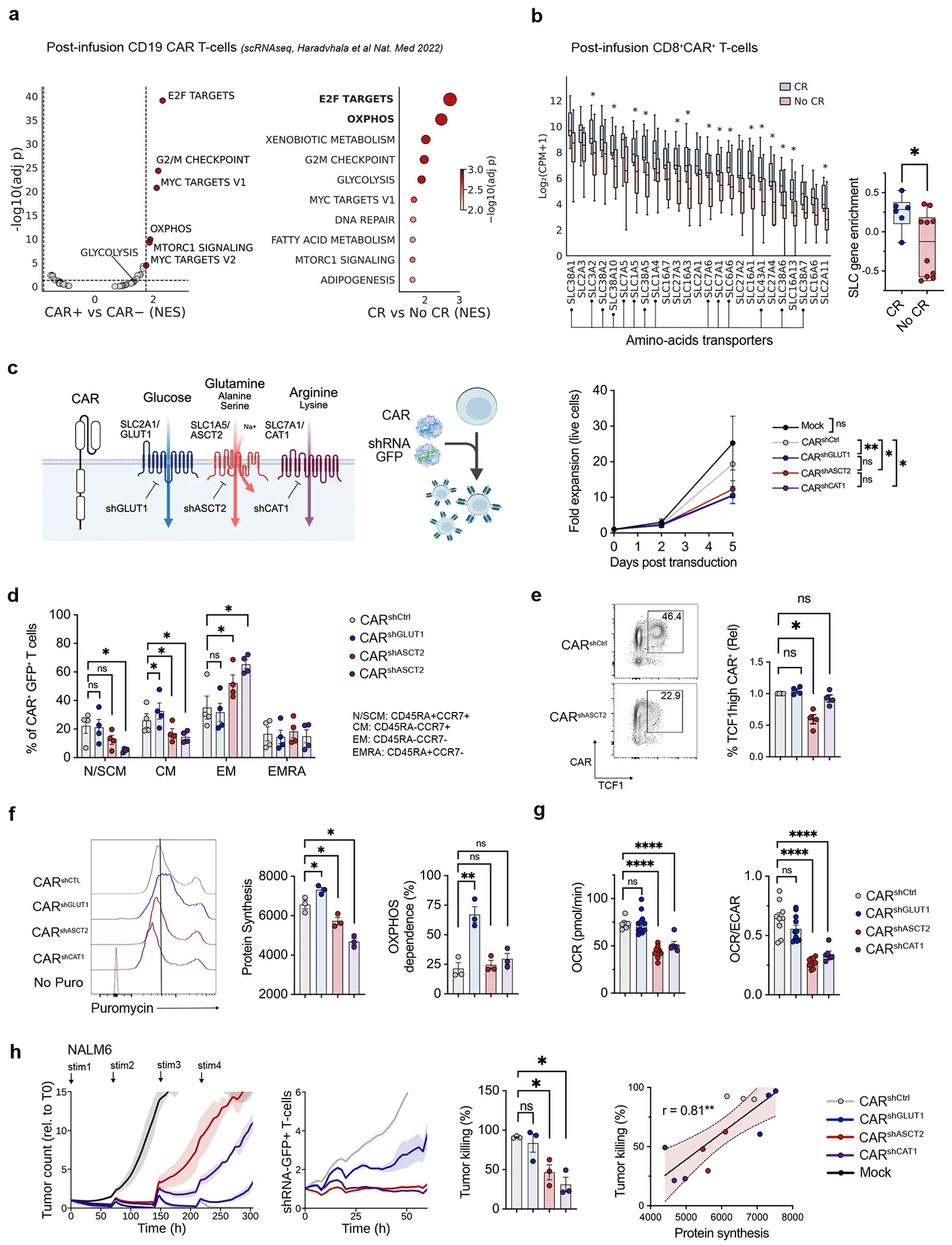
Amino acid transporters are required to sustain CAR T-cell cytotoxicity and preserve memory-like T-cell states. **a,** Analysis of publicly available scRNA-seq data from post-infusion CD19 CAR T cells (Haradhvala et al., *Nat. Med.*, 2022)^35^. Pseudo-bulk gene set enrichment analysis is shown as volcano plots of Hallmark pathways comparing CAR^+^ versus CAR^−^ T-cells (left), and CAR^+^ T-cells from patients achieving complete remission (CR) versus those not achieving complete remission (No CR; right). **b,** Expression of SLC transporters in post-infusion CAR T-cells according to clinical response. The displayed SLCs were annotated and selected based on reported cell-surface localization and nutrient transport functions (amino acids, monocarboxylates, glucose, and fatty acids). Expression in CD8^+^ CAR^+^ cells is shown at the individual gene level (log2(CPM + 1), left) and as an aggregated SLC gene set score (GSVA, right), comparing patients with CR (blue) and No CR (red). **c,** Experimental design: knockdown of major T-cell metabolite transporters by co-transduction with lentiviral particles carrying CAR and shRNAs targeting metabolite transporters. T-cell expansion was assessed across six donors in four independent experiments. **d,** Percentage of CAR T-cell subsets between shRNA conditions: naïve/stem cell memory (N/SCM, CD45RA+CCR7+), central memory (CM, CD45RA-CCR7+), effector memory (EM, CD45RA-CCR7−), effector memory RA-positive (EMRA, CD45RA+CCR7-). Data represent 4 donors from 2 independent experiments. **e,** Percentage of TCF1^high^ cells across shRNA-transduced CAR T-cells relative to the control condition (shCtrl) across shRNA conditions. Data represent four donors from two independent experiments. Representative flow cytometry plots for CAR^+^shCtrl^+^ and CAR^+^shASCT2^+^ cells are shown. **f,** Protein synthesis rate (puromycin incorporation) and OXPHOS dependence assessed by oligomycin treatment after 7 days of expansion across shRNA conditions. Data represent three independent donors. **g,** Oxygen consumption rate (OCR) and OCR/ECAR ratio measured in sorted GFP^+^CAR^+^ cells following 72h of co-culture with NALM6 cells. Data represent technical replicates from one representative donor. **h,** Incucyte cytotoxicity of CAR T cells expressing shRNAs co-cultured with NALM6-mKate^+^ cells at an effector-to-target ratio of 1:2 with four rounds of stimulation (indicated by arrows). Tumor cell counts (left) and GFP intensity of shRNA^+^ T-cells (middle) from one representative donor. Tumor growth was quantified as area under the curve (AUC) of tumor count (right). Corresponding tumor killing (%) was calculated relative to untransduced (Mock) T-cells. Data represent three independent donors. Statistical significance was determined using pseudo-bulk differential expression (DESeq2) and gene set enrichment (fgsea) with Benjamini–Hochberg correction in panel (a). In panel (b), GSVA enrichment scores were compared using two-tailed Welch’s *t*-tests. Two-tailed paired Student’s *t*-tests were used in panels (d–f), two-tailed unpaired *t*-tests were used in panel (g), and log-transformed AUC values were analyzed using two-tailed paired *t*-tests in panel (h). *P* values are indicated as follows: *P < 0.05, **P < 0.01, ***P < 0.001, and ****P < 0.0001; ns, not significant.

The profound amino acid perturbations accompanying CAR T-cell expansion and CRS led us to hypothesize that nutrient uptake regulators play a key role in sustaining CAR T-cell function following infusion. The capacity of cells to import extracellular nutrients is primarily governed by solute carrier (SLC) transporters—the largest family of membrane transporters^57,58^, which orchestrate metabolic adaptation to changes in nutrient availability and thereby regulate cellular fitness in nutrient-depleted environments^59,60^. To determine whether SLC expression associates with therapeutic responsiveness, we first analyzed a publicly available dataset of post-infusion CD19-targeted CAR T-cells from patients with R/R B-cell malignancies^35^ (**Extended Data Fig. 6a**). Compared to CAR^−^ T-cells, post-infusion CAR^+^ T-cells exhibited enrichment in pathways related to cell-cycle progression (E2F targets), MYC signaling, and mTORC1 activation. Consistent with the OXPHOS-shifted metabolic profile previously observed following *in vivo* CAR activation, OXPHOS-related genes were significantly upregulated, whereas glycolytic pathways remained unchanged. Importantly, cell-cycle and OXPHOS pathways were the two most prominently upregulated pathways in responders compared with non-responders (Fig. 5a).

Multiple SLCs mediating amino acid, glucose, monocarboxylate, and fatty acid transport were detected across post-infusion CD4^+^ and CD8^+^ CAR T-cell subsets (**Extended Data Fig. 6b**). Interestingly, analyzing these transporter profiles relative to clinical outcomes revealed a significant increase in 15 SLC transporters in CD8^+^ CAR T-cells from clinical responders compared to non-responders (Fig. 5b). Among these, 9 were explicitly dedicated to amino acid transport, with a strong emphasis on neutral amino acids (*SLC1A5/ASCT2*, *SLC38A5-6-10/SNAT5-6-10*) and cationic amino acids (*SLC7A1/CAT1*, *SLC7A6/ y^+^LAT2*). Notably, the major glucose transporters SLC2A1/GLUT1 and SLC2A3/GLUT3 showed no significant differential expression (Fig. 5b). In stark contrast to the CD8^+^ compartment, CD4^+^ CAR T–cells displayed no differentially expressed transporters between responders and non-responders (**Extended Data Fig. 6c**). These findings strongly implicate amino acid uptake, rather than glucose transport, as a critical determinant of CAR T-cell persistence and function *in vivo*.

To determine whether amino acid transporters play a distinct role in CAR T-cell function relative to glucose transporters, we attenuated expression of the alanine/serine/cysteine/glutamine transporter SLC1A5 (ASCT2), the arginine/cationic amino acid transporter SLC7A1 (CAT1), or the glucose transporter SLC2A1 (GLUT1) in CD19 CAR T-cells (Fig. 5c, **Extended Data Fig. 6d**). GLUT1 was selected as the comparator because it was previously implicated in CAR T-cell function^61,62^. shRNAs against these transporters markedly reduced surface expression of their respective target proteins (**Extended Data Fig. 6e–f**). Consistent with the established roles of SLCs in T-cell metabolism^51,53,63^, downregulation of ASCT2, CAT1, or GLUT1 impaired CAR T-cell expansion (Fig. 5d). However, because naïve (N) and stem-cell memory (SCM) T-cells preferentially rely on oxidative pathways^17,64^, we examined how individual transporter modulation impacted differentiation states. Attenuation of the amino acid transporters ASCT2 or CAT1—but notably not the glucose transporter GLUT1—caused a marked loss of memory-like N/SCM-like CD4^+^ and CD8^+^ CAR T-cells, with a corresponding increase in the effector memory (EM) fraction (Fig. 5d–5e, **Extended Data Fig. 6g**). These phenotypic changes were tightly coupled to shifts in global protein translation and oxidative capacity. Attenuation of either ASCT2 or CAT1 directly reduced overall protein synthesis rates (Fig. 5f) and decreased oxygen consumption rates (OCR) and OCR/extracellular acidification rate (ECAR) ratios following CAR activation, indicating impaired oxidative metabolism (Fig. 5f–5g). In contrast, GLUT1-downregulated cells maintained high protein synthesis rates that became strongly dependent on OXPHOS, showing no significant change in OCR following CAR activation. Most strikingly, these metabolic differences directly dictated long-term effector competence. Downregulation of ASCT2 or CAT1 caused a pronounced loss of cytotoxicity by round 2 of chronic antigen restimulation with CD19^+^ leukemia targets. Conversely, GLUT1-attenuated cells retained their full killing capacity through round 4 (Fig. 5h).

Because surface GLUT1 levels were only decreased by approximately 50% via shRNA knockdown (**Extended Data Fig. 6e**), this left open the possibility that residual expression or compensation by alternative glucose transporters masked a deeper glucose dependency. To discriminate between these mechanisms, we generated CRISPR/Cas9-mediated GLUT1 knockout (sgGLUT1) CAR T-cells (**Extended Data Fig. 6h**). Mirroring our findings of the knockdown approach, GLUT1 knock-out did not alter the T-cell differentiation phenotype (**Extended Data Fig. 6i–j**). Total protein synthesis rates remained fully comparable to control CAR T-cells, although translation shifted to become entirely dependent on OXPHOS (**Extended Data Fig. 6k**). Following CAR activation, baseline glycolysis was maintained—consistent with potential compensation by alternative glucose transporters—but the glycolytic reserve was drastically reduced (**Extended Data Fig. 6l**). Crucially, despite this glycolytic stress, GLUT1-knockout CAR T-cells retained full, unimpeded cytotoxic activity across multiple rounds of antigen restimulation (**Extended Data Fig. 6m**). Together, these results identify GLUT1 as the dominant mediator of glucose uptake in CAR T-cells, while demonstrating that amino acid transport is the principal metabolic driver sustaining OXPHOS, stemness, and durable cytotoxicity in CAR T-cells. These facilitated amino acid import mechanisms are expected to become increasingly vital when cells are forced to navigate the amino acid-depleted, nutrient-stressed systemic environments characteristic of clinical CRS (Fig. 4).

### Metabolic armoring with amino acid transporters boosts CAR T-cell efficacy

**Figure 6.**
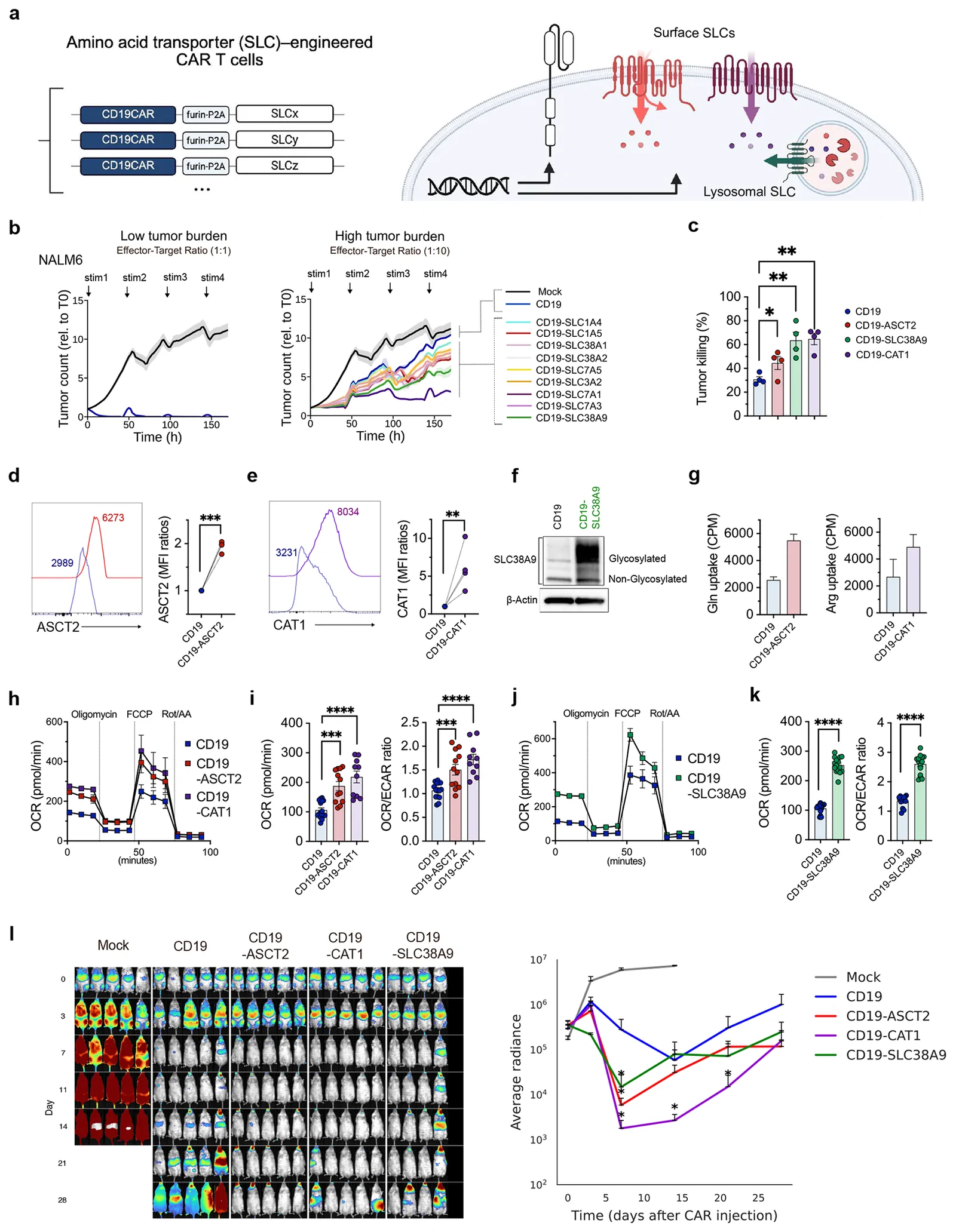
Metabolic armoring via amino acid transporter overexpression enhances CAR T-cell oxidative metabolism and anti-leukemic efficacy. **a,** Schematic of the genetic engineering strategy for amino acid transporter (SLC)-armored CAR T-cells. Bicistronic vectors encoding the CD19 CAR were generated to co-express amino acid transporters, including the surface transporters *SLC1A4, SLC1A5, SLC38A1, SLC38A2, SLC7A5, SLC7A1, SLC7A3* and *SLC3A2* as well as the lysosomal transporter *SLC38A9*. **b,** Incucyte cytotoxicity assay using NALM6-GFP^+^ target cells. At a high effector-to-target ratio (1:1), conventional CD19 CAR T-cells show complete tumor killing (left). At a low effector-to-target ratio (1:10), cytotoxic activity of CD19 CAR and CD19-SLC-armored CAR T-cells was compared under stress conditions across four rounds of stimulation by tracking tumor cell counts (right). Data show one representative donor. **c,** Quantification of the cytotoxicity screening shown in (b) comparing selected candidates (CD19BBζ-ASCT2, CD19-SLC38A9, and CD19-CAT1). Tumor growth was quantified by calculating the area under curve (AUC) of tumor counts across four consecutive rounds of stimulation at low E:T ratio (1:10). Corresponding tumor killing (%) was calculated relative to untransduced (Mock) T-cells. Data represent summary metrics across n = 4 independent donors. **d–e**, Cell surface expression of SLC1A5/ASCT2 and SLC7A1/CAT1 evaluated by flow cytometry using receptor-binding domain–derived reagents (RD114-RFP and BLV-rFC, respectively) in SLC-armored CAR T-cells at day 7 after initial stimulation. Representative histograms with mean fluorescence intensity (MFI) are shown. Surface expression was quantified as MFI ratios relative to the CD19 control (*n* = 4 independent donors). **f,** SLC38A9 expression in control and SLC38A9-armored CAR T-cells evaluated by immunoblotting. A representative blot from one donor with an actin loading control is shown. **g,** Glutamine and arginine uptake were measured in sorted CAR^+^ T-cells following 48 h co-culture with NALM6 leukemic cells. Uptake of [^3^H]-glutamine and [^3^H]-arginine was measured at room temperature for 10 min, and mean counts per minute (CPM) are shown. **h–k,** Metabolic flux analyses of control and SLC-armored CAR T-cells. Oxygen consumption rate (OCR) and OCR/ECAR ratios were measured in sorted CAR^+^ T-cells following 72 h co-culture with NALM6 leukemic cells. Seahorse measurements were recorded at baseline and after sequential treatment with oligomycin, FCCP, and rotenone/antimycin. **h,** Representative OCR profiles for CD19, CD19-ASCT2, and CD19-CAT1 CAR T-cells (*n* = 6–7 replicates per condition from one of two independent donors). **i,** Quantification of baseline OCR (left) and OCR/ECAR ratios (right) for CD19BBζ, CD19BBζ-ASCT2, and CD19BBζ-CAT1 CAR T-cells (*n* = 10–16 replicates from two independent donors). **j,** Representative OCR profiles for CD19 and CD19-SLC38A9 CAR T-cells (*n* = 9 replicates per condition from one of two independent donors). **k,** Quantification of baseline OCR (left) and OCR/ECAR ratios (right) for CD19 and CD19-SLC38A9 CAR T-cells (*n* = 15–16 replicates from two independent donors). **l,** *In vivo* anti-leukemic activity of control CD19 and SLC-armored CAR T-cells was evaluated in NSG mice engrafted with GFP^+^Luciferase^+^ NALM6 cells (1 × 10^6^) using a suboptimal limiting dose of CAR T-cells (0.75 × 10^6^). Representative bioluminescent images at the indicated time points are shown (left; *n* = 4–5 mice per group from one of two independent experiments). Tumor growth was quantified by bioluminescent radiance (right; *n* = 9–10 mice per group), and mean radiance is shown at the indicated time points. Statistical significance was determined using two-tailed paired *t*-tests in panels (c–e) and two-tailed unpaired Student’s *t*-tests in panels (i) and (k), and two-tailed unpaired Studenťs t-tests on log10-transformed values in panel (l). *P* values are indicated as follows: *P < 0.05, **P < 0.01, ***P < 0.001, and ****P < 0.0001; ns, not significant.

Based on the metabolic dependencies identified above, we next asked whether amino acid transport represents a limiting metabolic checkpoint for CAR T-cell function and whether enhancing nutrient acquisition through metabolic engineering could improve therapeutic efficacy. We selected candidate amino acid transporters based on their expression in post-infusion CAR T-cell scRNA-seq datasets and prior evidence supporting roles in amino acid uptake and sensing, T-cell metabolic fitness, and OXPHOS regulation^51–53,57,65–68^. To identify transporters capable of improving function under stress conditions, we generated a panel of metabolically armored (“MetaboArm”) CAR T-cells coexpressing individual transporters together with a CD19BBζ CAR construct, including the cell-surface transporters SLC1A4 (ASCT1), SLC1A5 (ASCT2), SLC38A1 (SNAT1), SLC38A2 (SNAT2), SLC7A5 (LAT1), SLC3A2 (4F2hc/CD98), SLC7A1 (CAT1), SLC7A3 (CAT3), and the lysosomal amino acid transporter SLC38A9 (Fig. 6a). MetaboArm CAR T-cells were subsequently evaluated in serial antigen-stimulation assays under stringent low effector-to-target (E:T) ratios of 1:10, conditions that mimic a high tumor burden. Whereas conventional CD19 CAR T-cells maintained tumor control through repeated restimulation at a 1:1 E:T ratio, reducing the ratio to 1:10 created a stringent challenge under which tumor control was rapidly lost (Fig. 6b), providing a sensitive platform to evaluate MetaboArm CAR T-cells. Comparative screening identified ASCT2, CAT1, and SLC38A9 as the most effective transporters for enhancing cytotoxic activity across independent donors (Fig. 6b–c). The top-performing MetaboArm CAR T-cell constructs were next characterized at the protein level. Surface overexpression of ASCT2 and CAT1 was confirmed by flow cytometry, whereas SLC38A9 protein overexpression was confirmed by immunoblotting (Fig. 6d–f). Functional radiotracer uptake assays demonstrated increased glutamine and arginine uptake by ASCT2- and CAT1-engineered CAR T-cells, confirming enhanced transporter activity following metabolic armoring (Fig. 6g).

The lead MetaboArm CAR constructs increased oxidative metabolism following CAR activation—reflected by elevated OCR and OCR/ECAR ratios—without a corresponding increase in glycolysis (Fig. 6h–k). To assess therapeutic efficacy *in vivo*, we challenged the constructs using a limiting dose of CAR T-cells (<1×10^6^ cells)^69^ in a leukemia model. The lead MetaboArm CAR constructs (ASCT2, CAT1, and SLC38A9) mediated a more rapid tumor clearance—occurring as early as day 3 post-infusion—and significantly improved sustained tumor control compared with conventional CD19 CAR T-cells (Fig. 6l). Together, these findings establish amino-acid transport as a dominant metabolic checkpoint regulating CAR T-cell fitness and demonstrate that metabolic armoring through transporter overexpression enhances antitumor efficacy. By equipping CAR T-cells to function within the amino-acid-restricted inflammatory environment that develops following infusion, this strategy offers a rational metabolic engineering approach to maximize durable clinical responses.

## DISCUSSION

Direct evaluation of CAR T-cell metabolism in patients remains challenging, leaving fundamental questions regarding the metabolic programs that sustain therapeutic responses unresolved. Using an integrated immuno-metabolic profiling framework combining clinical samples, longitudinal plasma metabolomics, and single-cell metabolic profiling, we identify a conserved post-infusion shift from glycolysis toward amino acid-supported OXPHOS in human CAR T-cells. Functional metabolic perturbation and transporter engineering further establish the mechanistic importance and therapeutic potential of this metabolic program. This amino acid-supported OXPHOS program was associated with stem-like CAR T-cell states, *in vivo* expansion, and complete clinical responses across independent CAR T-cell trials in pediatric and AYA patients with R/R B-ALL. Longitudinal metabolomic analyses further revealed that this metabolic remodeling was accompanied by progressive depletion of systemic amino acids during CAR T-cell expansion and CRS. Guided by these findings, we engineered metabolically armored CAR T-cells expressing amino acid transporters and demonstrated enhanced metabolic fitness and antitumor efficacy *in vivo*. These findings establish amino acid-supported OXPHOS as a central and targetable metabolic program in post-infusion CAR T-cells and reveal that successful therapeutic responses are accompanied by profound metabolic remodeling within both CAR T-cells and their host environment.

A major conceptual advance of this study is the demonstration that protein synthesis serves as an integrated functional readout of metabolic state in clinical CAR T-cell samples. Extending our recently established framework^37,70^, high-dimensional cytokine secretion dynamics revealed reproducible construct-specific CAR T-cell response trajectories that could be linked to metabolic and phenotypic states. Among all parameters evaluated, protein synthesis exhibited the strongest association with CAR T-cell functional outputs and emerged as a robust integrative metric linking metabolism, cytokine secretion, and cellular phenotype. Protein synthesis occupies a unique position at the interface of metabolism and immune function, acting both as a major consumer of cellular energy and as a central integrator of metabolic and signaling cues^39,40,46,71^. Moreover, translational control directly regulates the expression of key cytokines and transcription factors that drive T-cell differentiation and effector function, including IFNγ and T-bet^72,73,43,74,44,75,76^. While puromycin-based approaches such as SCENITH have primarily been used to classify metabolic pathway dependence, our findings indicate that protein synthesis itself can also function as a sensitive indicator of CAR T-cell state^41^. Consistent with a broader role for translational regulation in CAR T-cell function, recently identified engineering strategies including Regnase-1 and the pre–T-cell receptor α chain (pTα) have also been linked to post-transcriptional regulation and mRNA translation^77–80^. Because protein synthesis can be quantified at single-cell resolution using limited patient material, this approach enabled direct interrogation of CAR T-cell metabolism in clinical samples that would be difficult to evaluate using conventional metabolic assays.

Our findings also contribute to a growing shift in how T-cell metabolism is viewed *in vivo*. Classical models derived largely from *in vitro* activation systems emphasized glycolysis as the dominant metabolic program supporting T-cell activation and effector function. However, isotope-tracing studies in murine models have challenged this paradigm by demonstrating that physiologically activated T-cells utilize glucose differently *in vivo*, directing substantial carbon flux toward mitochondrial metabolism and anabolic pathways rather than predominantly toward lactate production^31,34^. More recent work further identified glutamine and acetate as major fuels supporting activated murine CD8^+^ T cells *in vivo*^33^. Together, these studies suggest that mitochondrial metabolism remains a central feature of physiologic immune responses and that conventional *in vitro* culture systems incompletely capture the metabolic programs operating during immune responses *in vivo*.

Whereas manufactured infusion products were overwhelmingly glycolysis dependent, circulating CAR T-cells rapidly acquired dependence on OXPHOS, glutamine uptake, and glutaminolysis following infusion. This metabolic transition was observed across distinct CAR constructs and independent clinical trials, suggesting that it represents a conserved feature of human CAR T-cell biology rather than a product-specific phenomenon. Single-cell analyses further demonstrated that OXPHOS^HighDep^ subsets were enriched within stem-like CAR T-cell populations associated with *in vivo* expansion and complete clinical response. These findings complement recent observations that long-lived memory CD8 T-cells generated following yellow fever vaccination rely heavily on oxidative metabolism^81^. They are also consistent with observations that responding CAR T-cell populations converge toward common metabolic states despite differences in CAR design.^21,26^ These findings indicate that CAR T-cell metabolism is dynamically remodeled following infusion and reflects adaptation to the metabolic landscapes encountered in patients rather than solely cell-intrinsic programs established during manufacturing.

A striking finding from our longitudinal plasma metabolomic analyses was the substantial depletion of multiple amino acids, including glutamine and arginine, during the period of peak CAR T-cell expansion and CRS. Similar alterations have been reported in diverse inflammatory conditions, including graft-versus-host disease, COVID-19, and hyperinflammatory syndromes, but their significance during CAR T-cell therapy has remained poorly understood^82,27–29,83,30^. By coupling serial cytokine and metabolite measurements within individual patients, we found a close temporal association between inflammatory cytokine production and amino acid depletion, supporting a substantial immune-driven contribution to this metabolic remodeling. These observations suggest that CAR T-cells do not operate within static nutrient conditions but instead adapt to metabolic constraints that evolve during therapy. More broadly, our findings support a model in which CAR T-cell state is shaped not only by intrinsic signaling programs established during manufacturing, but also by the dynamic metabolic landscape encountered in patients. Consistent with this concept, recent studies have shown that systemic metabolic perturbations during critical illness, as well as changes in nutrient availability, can remodel human T-cell metabolism and therapeutic T-cell function^56,84–86^. Supporting the functional importance of this metabolic environment, nutritional interventions such as β-hydroxybutyrate supplementation or postprandial lipid remodeling can enhance CAR T-cell oxidative metabolism and antitumor activity^86,87^.

The emergence of amino acid depletion during peak CAR T-cell expansion identified nutrient acquisition as a key determinant of CAR T-cell adaptation *in vivo*. Consistent with this hypothesis, reanalysis of published post-infusion CAR T-cell datasets revealed enrichment of amino acid transporter transcripts within responder-associated CAR T-cell populations^35^. This observation aligns with seminal studies demonstrating that antigen receptor signaling coordinates amino acid transporter expression with metabolic reprogramming to sustain mTOR signaling, biosynthesis, and proliferation^88–91,53,57^. Functionally, disruption of amino acid uptake impaired protein synthesis, oxidative metabolism, maintenance of stem-like subsets, and durable cytotoxicity. These findings identify amino acid transport as a critical metabolic bottleneck limiting CAR T-cell adaptation to the post-infusion environment.

Recent studies have demonstrated that metabolic engineering of nutrient acquisition can enhance CAR T-cell function^61,85,92–96^, including through GLUT1 or GLUT3 armoring ^61,62,85,94,96^. Our findings extend this concept by demonstrating that the benefits of nutrient transporter engineering are highly context dependent and that glucose and amino acid transport do not contribute equally to CAR T-cell fitness. In our studies, GLUT1 disruption profoundly rewired CAR T-cell metabolism, abolishing glycolytic capacity and markedly increasing OXPHOS dependence, yet exerted only modest effects on sustained cytotoxicity compared with perturbation of amino acid transport. Similarly, analysis of published post-infusion CAR T-cell datasets revealed stronger enrichment of OXPHOS-associated transcriptional programs than glycolytic programs in responding patients. Together, these findings support a model in which mitochondrial metabolism, rather than maximal glycolytic flux, represents a dominant metabolic requirement for CAR T-cell function following infusion.

*In vivo* CRISPR screens have recently demonstrated that the factors governing CAR T-cell expansion and persistence vary substantially according to the environment, while SLC-focused genetic screens have shown that the importance of specific nutrient transporters depends on local nutrient availability.^97–100^ Importantly, the transporters identified here emerged from metabolic adaptations observed in patient CAR T-cells rather than from *a priori* optimization under defined culture conditions, highlighting the value of patient-derived metabolic states for guiding CAR engineering strategies. Building on this concept, we systematically evaluated a panel of amino acid transporters and identified SLC1A5, SLC7A1, and SLC38A9 as effective engineering targets. While amino acid transport has long been recognized as a critical regulator of T-cell activation and mTORC1 signaling, direct comparison of multiple transporter families within a common CAR engineering framework has been limited^92,101^. The identification of SLC7A1 highlights the importance of arginine acquisition during CAR T-cell responses, consistent with the established role of arginine in sustaining T-cell fitness and antitumor activity^13^. Interestingly, Huang et al. reported that loss of SLC7A1 or SLC38A2 altered the balance between memory and effector differentiation through reduced mTORC1 signaling in murine CD8^+^ T-cells following acute LCMV infection^97^. In our studies, perturbation of SLC7A1 and SLC1A5 also altered the relative distribution of naïve, memory, and effector populations in human CAR T-cells, although with distinct differentiation patterns. Together, these findings highlight the context-dependent relationship between amino acid transport, metabolic signaling, and T-cell differentiation.

Consistent with the established role of SLC1A5 in supporting T-cell activation and metabolic fitness^89,102,103^, Navarro et al. recently showed that SLC1A5 armoring enhanced BCMA CAR T-cell efficacy in multiple myeloma under conditions of limited glutamine availability.^101^ The emergence of SLC38A9 was particularly intriguing because, unlike conventional plasma membrane transporters, SLC38A9 functions as a lysosomal arginine transporter and amino acid sensor that directly couples nutrient availability to mTORC1 activation^66,67^. Its ability to enhance CAR T-cell fitness therefore suggests that optimizing nutrient sensing, in addition to nutrient uptake, may improve function under nutrient-restricted conditions. Together, these findings support a model in which effective metabolic engineering strategies should reflect the nutrient dependencies imposed by specific physiological and disease environments rather than relying on a universal metabolic solution.

In summary, we establish a patient-derived metabolic framework defining how human CAR T cells adapt following infusion and identify amino acid-supported OXPHOS as a conserved metabolic program associated with expansion, persistence, and clinical response. By linking clinical metabolic profiling to functional perturbation and transporter engineering, these studies provide mechanistic insight into human CAR T-cell biology and establish a rationale for the development of metabolically armored cellular therapies designed to function within nutrient-restricted environments.

## METHODS

### Cell lines

NALM6-GL cells engineered to express GFP and luciferase reporters were obtained from Dr. S. Grupp (University of Pennsylvania). NALM6-GL-CD33^+^ cell line was derived from NALM6-GL by stable lentiviral transduction of a human CD33 expression vector. Surface expression of CD19, CD22, and CD33 was quantified using the Quantibrite PE kit (BD Biosciences) according to the manufacturer’s instructions, yielding 1.00 × 10^5^, 3.28 × 10^4^, and 1.65 × 10^5^ molecules per cell, respectively. HEK293T and parental NALM6 human B-cell acute lymphoblastic leukemia cells were purchased from ATCC. A NALM6-mKate2^+^ derivative was generated by stable lentiviral transduction of an mKate2 expression vector. NALM6 cells were maintained in RPMI 1640 medium supplemented with 10% (v/v) fetal bovine serum (FBS), 1% GlutaMax, and 1% penicillin/streptomycin (complete RPMI 1640). HEK293T cells were maintained in DMEM supplemented with 10% (v/v) FBS, 1% GlutaMax, 1% pyruvate, 1% HEPES, and 1% penicillin/streptomycin (complete DMEM). All cell lines were confirmed to be free of Mycoplasma contamination using the MycoAlert Detection Kit (Lonza, Basel, Switzerland).

### Mice

NOD.Cg-Prkd^scid Il2r^tm1Wjl/SzJ (NSG) mice were bred in-house and maintained at the National Institutes of Health under specific pathogen-free (SPF) conditions. Animals were housed on a 12-h light/dark cycle corresponding to daylight hours in Bethesda, MD, USA. Mice aged 6–8 weeks were used; both males and females were included. All experiments were performed under protocols approved by the NCI Institutional Animal Care and Use Committee (IACUC).

### Human peripheral T-cells from healthy donors

Peripheral blood mononuclear cells (PBMCs) from healthy adult donors were obtained from the Department of Transfusion Medicine, NIH Clinical Center, under IRB-approved protocols. T cells were enriched using the RosetteSep Human T Cell Enrichment Cocktail (STEMCELL Technologies) with Ficoll-Paque PLUS (GE Healthcare), according to the manufacturer’s instructions. Purified T-cells were cryopreserved at 20–50 × 10^6 cells per vial in FBS containing 10% DMSO (Sigma-Aldrich) until CAR T-cell manufacturing.

### Patient-derived CAR T-cell samples

Cryopreserved CAR T-cell infusion products were obtained from pediatric, adolescent, and young adult patients with B-cell acute lymphoblastic leukemia (B-ALL) enrolled in NCI CAR T-cell clinical trials (CD19/CD22 bispecific CAR trials, NCT03448393/ NCT05098613); CD22 CAR trial, NCT02315612) following GMP manufacturing. Post-infusion peripheral blood mononuclear cells (PBMCs) were collected 7–14 days after infusion; in the CD19/CD22 CAR trials, PBMCs were processed and analyzed fresh within 3 hours of collection, whereas in the CD22 CAR trial, PBMCs were cryopreserved prior to analysis. In parallel, plasma samples were collected longitudinally between day 0 and day 28 after infusion and cryopreserved for metabolomic and cytokine analyses. All patient-derived samples were obtained under NIH IRB–approved protocols with informed consent.

## METHOD DETAILS

### Generation of lentiviral vectors and virus production

The lentiviral vectors expressing second-generation CAR constructs contained anti-human CD19, CD22, or CD33 scFvs (clones FMC63, m971, and Hu195, respectively). For 4-1BB–based CARs, a CD8α hinge–transmembrane domain with a 4-1BB costimulatory signaling domain was included, whereas CD28-based CARs contained a CD28 hinge–transmembrane domain with a CD28 costimulatory signaling domain. All constructs incorporated the signaling motif of the CD3ζ chain and were expressed under the control of the human EF1α promoter. Lentiviral bicistronic vectors containing CAR and SLC overexpression sequences were based on second-generation CAR backbones, incorporating a Furin–P2A cassette followed by codon-optimized SLC open reading frames (ORFs) derived from UNIPROT human SLC amino acid sequences. Lentiviral vectors expressing shRNAs along with an EGFP reporter and targeting SLC1A2/GLUT1, SLC1A5/ASCT1, SLC7A1/CAT1, or a non-targeting control were used as previously described. Lentiviral batches were produced by transfecting HEK293T cells with third-generation packaging plasmids (pMDLg/pRRE, pRSV-Rev, and pMD2.G; Addgene) together with the transfer plasmid using Lipofectamine 3000 (ThermoFisher Scientific) or polyethyleneimine (PEI). Viral supernatants were collected at 24 and 48 hours post-transfection, filtered through 0.45 μm membranes, layered over sucrose, and concentrated by ultracentrifugation. Viral titers were determined by functional titration based on jurkat cell transduction.

### Laboratory-grade CAR T-cell manufacturing

Human T cells were isolated from PBMCs using the RosetteSep Human T Cell Enrichment Cocktail (STEMCELL Technologies) according to the manufacturer’s instructions. T cells were stimulated with Dynabeads Human T-Expander (ThermoFisher Scientific) at a 1:2 (T cell:bead) ratio in AIM-V medium supplemented with 5% (v/v) FBS, 1% (v/v) GlutaMAX, 1% (v/v) HEPES, and 1% (v/v) penicillin/streptomycin (complete AIM-V), together with recombinant human IL-2 (40 IU/mL), for 36 hours. Activated T cells were transduced with concentrated lentiviral vectors in the presence of protamine sulfate (10 μg/mL) and human IL-2 (200 IU/mL) on days 2 and 3 by spin infection (1,000 g, 32 °C, 2 hours). On day 4, Dynabeads and residual viral supernatants were removed, and transduced T cells were transferred into fresh complete AIM-V supplemented with human IL-2 (200 IU/mL). Cells were subsequently passaged every 2 days in complete AIM-V with human IL-2 (200 IU/mL). CAR T-cell transduction efficiency was assessed on days 7–8 prior to use in functional assays.

### Flow cytometry

Cells were stained with fluorochrome-conjugated antibodies using assay-specific staining panels and conditions. Where indicated, intracellular staining was performed following fixation and permeabilization using the FOXP3/Transcription Factor Staining Buffer Set according to the manufacturer’s instructions. For detection of CAR expression, patient samples were analyzed using a CD19 detection reagent (Miltenyi Biotec) for CD19 and CD19/CD22 CARs, or an anti-m971 scFv antibody for CD22 CARs. For laboratory-generated CAR T-cells, CAR expression was assessed using Protein L, a CD19 detection reagent (Miltenyi Biotec), or an anti-Whitlow antibody, depending on the construct and experiment. Samples were acquired on BD LSRFortessa, ThermoFisher Attune NxT, or Cytek Aurora flow cytometers, depending on the experiment. Spectral flow cytometry data were unmixed using SpectroFlo software (Cytek Biosciences). Downstream analyses were performed using FlowJo software (BD Biosciences) and, for spectral flow cytometry datasets, custom Python pipelines for arcsinh normalization, dimensionality reduction, PARC-based clustering (Levin et al 2021), and cluster-level quantitative analyses.

### Immunotron-based cytokine bead array

Cytokine secretion dynamics were quantified using the IMMUNOtron robotic platform as previously described. Human CAR T cells were co-cultured with NALM6 leukemic target cells in 96-well plates at multiple effector-to-target (E:T) ratios in 200 μL complete RPMI medium. Following co-culture plating, plates were briefly centrifuged to promote cell contact and immediately loaded onto the TECAN-based automated platform to enable longitudinal supernatant collection. At defined time points following co-culture initiation (1, 3, 6, 12, 18, 24, 30, 36, 42, 48, 60, and 72 h), 20 μL of culture supernatants were automatically collected with minimal perturbation of cell pellets and replenished with 20 μL of fresh medium. Collected supernatants were stored at −20°C until analysis. Cytokine concentrations were quantified using multiplex human cytokine bead array (CBA) assays (BD Biosciences) according to the manufacturer’s instructions. Briefly, thawed supernatants were incubated with capture beads and detection reagents, followed by flow cytometric acquisition on a BD Fortessa cytometer.

### Metabolic flux and functional CAR T-cell assays

CAR T-cells were activated by co-culture with NALM6 cells in complete RPMI 1640 medium at an effector-to-target ratio of 1:2, using 1 × 10^6^ CAR^+^ T-cells and 2 × 10^6^ NALM6 cells per well in 6-well plates. Cultures were maintained for 72 hours, with 50% of the medium replaced with fresh complete RPMI 1640 at 48 hours.

### Seahorse metabolic flux analysis

Oxygen consumption rates (OCR) and extracellular acidification rates (ECAR) were measured using a Seahorse XFe96 Analyzer (Agilent). Calibration plates were hydrated overnight at 37 °C with XF calibrant (Agilent). Culture plates were coated with Cell-Tak (22.4 μg/mL), washed twice with water, air-dried, and stored at 4 °C until use. Following co-culture with tumor cells, the absence of residual GFP^+^ target cells was confirmed by flow cytometry, and CAR T-cells were resuspended in Seahorse XF RPMI assay medium supplemented with glucose, pyruvate, and glutamine. A total of 2 × 10^5^ cells per well were plated in Cell-Tak–coated assay plates and incubated at 37 °C for 1 hour in a non-CO_2_ incubator. OCR and ECAR were subsequently measured at 37 °C using the Seahorse XF Cell Mito Stress Test kit (Agilent), following sequential injections of oligomycin (2 μM), FCCP (1 μM), and rotenone/antimycin A (1 μM).

### Mitochondrial mass and polarization

Mitochondrial biomass was assessed in a total volume of 50 μls by MitoTrackerGreen or MitoTrackerDeepRed staining (20 nM; Invitrogen, Molecular Probes), while mitochondrial transmembrane potential levels were monitored by staining with MitoTrackerRed (50 nM; Invitrogen, Molecular Probes). Incubations were performed in the dark for 20 minutes in PBS + 2% FBS at RT. Samples were acquired on BD LSRFortessa or ThermoFisher Attune NxT flow cytometers.

### Metabolite transporter surface expression with receptor-binding domains (RBDs) Nutrient uptake assays

Sorted CAR^+^ T-cells (5 × 10^5^ cells/ condition), activated by coculture with NALM6, were washed twice with HBSS (400g, 4 min, room temperature). Cells were then starved of arginine or glutamine for 30 min at 37°C in 30 μL of RPMI medium lacking the respective nutrient (US Biological). Uptake assays were performed by adding radiolabeled substrates at a final concentration of 2 μM (0.5 μCi in a total reaction volume of 50 μL): L-[3,4-^3^H]-arginine monohydrochloride or L-[3,4-^3^H(N)]-glutamine (Perkin Elmer). Following a 10-min incubation at room temperature, cells were washed twice with cold PBS containing 2% FBS (1,000g, 3 min) and lysed in 500 μL of 0.1% SDS. Incorporated radioactivity was measured in a Hidex 300 SL liquid scintillation counter (in a total volume of 4.5 mL scintillation fluid (Perkin Elmer)).

### Protein-synthesis–based single-cell metabolic assays

Protein synthesis was measured by incubating cells with puromycin (10 μg/mL) for 30 min in complete RPMI 1640 medium in 96-well plates. After incubation, cells were washed twice with PBS containing 2% FBS, stained with fluorochrome-conjugated surface marker antibodies and CAR detection reagents, and subsequently fixed and permeabilized using the FOXP3/Transcription Factor Staining Buffer Set (eBioscience) according to the manufacturer’s instructions. Puromycin incorporation was detected by intracellular staining with anti-puromycin antibody.

### Metabolic dependencies for protein synthesis

For metabolic dependency experiments, cells were resuspended in complete RPMI 1640, divided into separate wells, and pre-treated for 15 min with DMSO (vehicle), 2-deoxy-D-glucose (2DG, 150 mM), oligomycin (1 μM), V-9302 (20 μM), CB-839 (15 μM), or etomoxir (4 μM). Puromycin (10 μg/mL) together with the corresponding drug was then added in complete RPMI 1640 for an additional 30 min. Background controls included treatment with 2DG plus oligomycin (DGO) and/or cycloheximide (CHX, 100 μg/mL), as well as no-drug/no-puromycin (No Puro) conditions. Metabolic dependencies were calculated as previously described using puromycin mean fluorescence intensities (MFI): [(Puro-MFI^DMSO^- PuroMFI^DRUG^)/ (Puro-MFI^DMSO^- PuroMFI^DGO/CHX^)]*100.

### Spectral flow cytometry–based metabolic dependency assays

Assessment of metabolic dependencies for protein synthesis was adapted to high-dimensional spectral flow cytometry. Cells were divided into DMSO, oligomycin (OLIGO), cycloheximide (CHX), and no-puromycin (No Puro) treatment conditions. After incubation, cells were washed twice with PBS containing 2% FBS and incubated for 20 min at 37 °C with antibodies against chemokine receptors (CCR4, CCR5, CCR7, CXCR3). Cells were then incubated for 20 min at room temperature with additional antibodies against surface markers (CAR detection reagent, CD3, CD4, CD8, CD25, CD69, CD71, CD45RA, CD45RO, CD62L, CD127, CD27, CD28, 4-1BB, ICOS, CD95, PD-1, TIM-3, LAG-3, CD39) and fixable viability dye. Following washes in PBS containing 2% FBS, cells were fixed and permeabilized as described earlier and subsequently stained overnight (16 h) with antibodies against intracellular markers (TCF1, HELIOS, T-bet) in fixation/permeabilization buffer. The next day, cells were stained with anti-puromycin antibody for 45 min in fixation/permeabilization buffer, washed with PBS containing 2% FBS, and analyzed on a 5-laser Cytek Aurora spectral flow cytometer (Cytek Biosciences, Fremont, CA). Data were unmixed using SpectroFlo software (Cytek Biosciences, Fremont, CA), and live CD3^+^CAR^+^ cell populations were gated and annotated by sample ID and drug condition in FlowJo (BD Biosciences, Ashland, OR) before export for downstream analysis in Python v3.12.7. Median fluorescence intensity (MFI) values were normalized using a dynamic arcsinh transformation, after which all cells were concatenated and clustered with the PARC algorithm (Phenotyping by Accelerated Refined Community-partitioning) based on all markers excluding puromycin staining. Then, metabolic dependencies for OXPHOS were calculated within each cluster/sample condition, as described earlier.

### Plasma metabolomic analyses

Targeted metabolomics was performed on human plasma using stable isotope dilution LC/MS-MS based targeted multiple reaction monitoring (MRM) assays. Metabolites were extracted by adding 80 ul of ice-cold methanol to 20 ul of plasma followed by centrifugation at 14,000 g for 10 min. The extracted metabolite solution was divided into 4 tubes, and dried in SpeedVac^®^ vacuum concentrator (ThermoFisher Scientific, Waltham, MA) and stored in −80 °C. Selected metabolites in the glycolysis, pentose phosphate, TCA cycle, oxidative phosphorylation pathways were measured and quantified. In addition, selected purine and pyrimidine and short chain fatty acids were measured. The LC-MS analysis was performed on Thermo TSQ^™^ Quantiva triple quadrupole mass spectrometers (Thermo Scientific, San Jose, CA) coupled to a NexeraXR LC system (Shimadzu Scientific Instruments, Columbia, MD). Both the HPLC and mass spectrometer were controlled by Xcalibur^™^ software (Thermo Scientific). Quantitation of all metabolites was carried out using Xcalibur^™^ Quan Browser (Thermo Scientific). Calibration curves for each metabolite were constructed by plotting the ratio of the reference compound over the spiked in isotopic standard peak area ratios obtained from the calibration standards curve and fitting the data using linear regression with 1/*X* weighting. The metabolite concentrations in samples were then interpolated using the linear function obtained from the calibration curve.

### Analysis of publicly available scRNA-seq datasets

Publicly available scRNA-seq data from Haradhvala et al. Nature Medicine 2022 (GEO accession: GSE197268)^35^ were downloaded and re-analyzed. Raw sequencing data were processed using the NIH CCBR SINCLAIR scRNA-seq pipeline, including quality control, low-quality cell and doublet filtering, normalization, and Seurat-based data integration. Post-infusion day 7 samples, including CAR-positive and CAR-negative T-cell samples, were selected for downstream analyses. Original cell annotations and clinical response information were shared by the authors. Pseudo-bulk CPM-normalized gene expression matrices were generated at the sample level for downstream comparisons, and differential expression analyses were performed on aggregated expression matrices, followed by Hallmark pathway enrichment analyses using MSigDB gene sets with Benjamini–Hochberg correction for multiple testing. In parallel, solute carrier (SLC) genes were manually annotated based on known cell surface localization and substrate specificity, including amino acids, glucose, lipids, and monocarboxylates. Based on this annotation framework, a manually curated SLC nutrient transporter gene set was generated and used for GSVA-based pathway analyses.

### Statistical analyses

Statistical analyses were performed using GraphPad Prism 9.4.1 (GraphPad Software), R (v4.3.1) with Seurat (v5.0.1) or custom Python notebooks using NumPy, pandas, SciPy, and statsmodels unless otherwise indicated. The number of biological replicates, patients, donors, mice, or independent experiments is indicated in the corresponding figure legends. For in vitro studies using healthy donor–derived CAR T-cells, individual donors were considered biological replicates. For patient-derived analyses, each patient sample represented an independent biological replicate. For in vivo experiments, individual mice were considered biological replicates.

Comparisons between two groups were performed using two-tailed paired or unpaired Student’s t tests as appropriate. Welch’s correction was applied when variances were unequal. Nonparametric paired comparisons were performed using Wilcoxon matched-pairs signed-rank tests. Correlation analyses were performed using Pearson correlation coefficients unless otherwise specified. Statistical details for each experiment, including statistical tests, n values, and significance thresholds, are provided in the corresponding figure legends.

For metabolomic, cytokine, and high-dimensional cytometry analyses involving multiple comparisons, false discovery rate (FDR) correction was performed using the Benjamini–Hochberg method where indicated. Statistical significance was defined as follows: *p/q < 0.05, **p/q < 0.01, ***p/q < 0.001, and ****p/q < 0.0001.

## Supplementary Material

This is a list of supplementary files associated with this preprint. Click to download.

- Moralyetal20260824ExtendedData.pdf

## Figures and Tables

**Table:** REAGENTS TABLE

REAGENT or RESOURCE	SOURCE	IDENTIFIER
Antibodies		
Anti-human CD3-PE-Cy5 (clone UCHT1), 1:100	BioLegend	Cat#300410; RRID: AB_314064
Anti-human CD4-Alexa Fluor 700 (clone SK3), 1:100	BioLegend	Cat#344622; RRID: AB_2563150
Anti-human CD8-Spark YG 593 (clone QA18A37), 1:100	BioLegend	Cat#303808; RRID: AB_2904332
Anti-human Whitlow/218 Linker Alexa Fluor 647 (clone E3U7Q), 1:100	Cell Signaling	Cat#69310L; RRID: AB_3626306
Anti-human CD25-BUV496 (clone 2A3), 1:200	BD Biosciences	Cat#612918; RRID: AB_2870203
Anti-human CD69-BUV805 (clone FN50), 1:200	BD Biosciences	Cat#748763; RRID: AB_2857327
Anti-human CD71-RB780 (clone M-A712), 1:200	BD Biosciences	Cat#755839; RRID: AB_3688144
Anti-human CD45RA-BV650 (clone HI100), 1:200	BioLegend	Cat# 304136; RRID: AB_2563653
Anti-human CD45RO-BV570 (clone UCHL1), 1:200	BioLegend	Cat#304226; RRID: AB_2563818
Anti-human CD197(CCR7)-BUV615 (clone 2-L1-A),	BD Biosciences	Cat#751099; RRID: AB_2875131
Anti-human CD62L-BUV496 (clone DREG56)	Thermo Fischer Scientific	Cat#364-0629-42; RRID: AB_2925341
Anti-human CD127(IL7R)-Pacific Blue (clone A019D5)	BioLegend	Cat#351306; RRID: AB_10718639
Anti-human CD27-APC-Fire 810 (clone O323)	BioLegend	Cat#302864; RRID: AB_2894450
Anti-human CD28-BUV737 (clone CD28.2)	BD Biosciences	Cat#612815; RRID: AB_2870140
Anti-human CD137(4-1BB)-PE-Dazzle 594 (clone 4B4-1)	BioLegend	Cat#309826; RRID: AB_2566260
Anti-human CD278(ICOS)-BV711 (clone DX29)	BD Biosciences	Cat#563833; RRID: AB_2738440
Anti-human CD95-BB700 (clone DX2)	BD Biosciences	Cat#566543; RRID: AB_2869780
Anti-human CD279(PD1)-BV605 (clone NAT105)	BioLegend	Cat#367426; RRID: AB_2721545
Anti-human CD366(TIM3)-BV750 (clone F38-2E2)	BioLegend	Cat#345056; RRID: AB_2892420
Anti-human CD223(LAG3)-PE-Fire700 (clone 7H2C65)	BioLegend	Cat#369226; RRID: AB_3106034
Anti-human CD39-APC-Cy7 (clone A1)	BioLegend	Cat#328226; RRID: AB_2571981
Anti-human CD183(CXCR3)-BUV661 (clone 1C6/CXCR3)	BD Biosciences	Cat#741649; RRID: AB_2871046
Anti-human CD194(CCR4)-BV421 (clone L291H4)	BioLegend	Cat#359414; RRID: AB_2562435
Anti-human CD195(CCR5)-BV785 (clone 3A9)	BD Biosciences	Cat#565001; RRID: AB_2739039
Anti-human TCF1(TCF7)-PE (clone 7F11A10)	BioLegend	Cat#655208; RRID: AB_2728492
Anti-human T-bet-BUV395 (clone 04-46)	BD Biosciences	Cat#568109; RRID: AB_3684041
Anti-human HELIOS-PE-Cy7 (clone 22F6)	BioLegend	Cat#137236; RRID: AB_2565990
Anti-puromycin-Alexa Fluor 488 (clone 2A4)	Jonathan Yewdell, NIAID, USA	RRID: AB_2619605
Anti-puromycin-Alexa Fluor 488 (clone 2A4)	BioLegend	Cat#381506; RRID: AB_2927825
Anti-puromycin-Alexa Fluor 647 (clone 2A4)	Jonathan Yewdell, NIAID, USA	RRID: AB_2619605
Anti-puromycin-Alexa Fluor 647 (clone 2A4)	BioLegend	Cat#381508; RRID: AB_2927826
Anti-human CD3-APC-Cy7 (clone SK7)	BD Biosciences	Cat# 557832; RRID: AB_396890
Anti-human CD4-PerCP-Cyanine5.5 (clone SK3)	BioLegend	Cat#344608; RRID: AB_1953236
Anti-human CD8-BV605 (clone HIT8a)	BioLegend	Cat#300936; RRID: AB_2832575
Bacterial and virus strains		
One Shot Stbl3 Chemically Competent E. coli	ThermoFisher Scientific	C737303
Biological samples		
Human peripheral blood mononuclear cells	NIH Blood Bank	N/A
Patient-derived CAR T-cell pre-infusion products	NIH Pediatric Oncology Branch	N/A
Patient-derived post-infusion peripheral blood mononuclear cells	NIH Pediatric Oncology Branch	N/A
Patient-derived post-infusion plasma samples	NIH Pediatric Oncology Branch	N/A
Chemicals, peptides, and recombinant proteins		
Recombinant human IL-2 (Aldesleukin)	Novartis	0078-0495-61
CD19 CAR Detection Reagent, human	Miltenyi Biotec	130-129-550
Recombinant Human Siglec-2/CD22 Fc Alexa Fluor^®^ 647 Protein	R&D SYSTEMS	AFR1968-020
Purified Recombinant Biotinylated Protein L	ThermoFisher Scientific	29997
Puromycin	Millipore Sigma	P4512
Oligomycin	Millipore Sigma	O4876
2-Deoxy-D-glucose (2DG)	Millipore Sigma	D8375
V-9302	Selleckchem	S8818
CB-839	Selleckchem	S7655
Etomoxir	Selleckchem	S8244
Cycloheximide	Millipore Sigma	239764
Streptavidin-Alexa Fluor647	ThermoFisher Scientific	S32357
LIVE/DEAD Fixable Blue Dead Cell Stain Kit	ThermoFisher Scientific	L34962
LIVE/DEAD Fixable Violet Dead Cell Stain Kit	ThermoFisher Scientific	L34964
GLUT1 RBD-GFP	Metafora biosystems	Glut1-G100
ASCT2 RBD-RFC	Metafora biosystems	N/A
CAT1 RBD-rFc	Metafora biosystems	N/A
MitoTrackerGreen FM	ThermoFisher Scientific	M7514
Tetramethylrhodamine, Methyl Ester, Perchlorate (TMRM)	ThermoFisher Scientific	T668
MitoSOX	ThermoFisher Scientific	M36008
Critical commercial assays		
RosetteSep^™^ Human T Cell Enrichment Cocktail	STEMCELL Technology	15061
Dynabeads Human T-Activator CD3/CD28 for T Cell Expansion and Activation	ThermoFisher Scientific	11131D
Seahorse XF Cell Mito Stress Test Kit	Agilent	103015-100
MycoAlert Mycoplasma Detection Kit	Lonza	LT07-318
Human Th1/Th2/Th17 CBA Kit	BD Biosciences	560484
Human Chemokine CBA Kit	BD Biosciences	552990
Quick Start Bradford Protein Assay Kit	Bio-Rad	5000201
eBiosciences FOXP3/Transcription Factor Staining Buffer Set	Invitrogen	00-5523-00
Quantibrite PE Phycoerythrin Fluorescence Quantification Kit	BD Biosciences	340485
Experimental models: Cell lines		
Human: HEK293T	ATCC	Cat#CRL-3216; RRID: CVCL_0063
Human: NALM6-GL	Stephan Grupp, U. Penn, USA	N/A
Human: NALM6-mKate2	This paper	N/A
Human: NALM6-GL-CD33^+^	This paper	N/A
Human: JURKAT	IGMM, France	N/A
Experimental models: Organisms/strains		
Mouse: NSG: NOD.Cg-Prkdc^scid^Il2rg^tm1Wjl^/SzJ	The Jackson Laboratory	Strain#:005557; RRID:IMSR_JAX:005557
Recombinant DNA		
Plasmid: pMDLg/pRRE	Didier Trono, EPFL, Switzerland	RRID:Addgene_12251
Plasmid: pRSV-Rev	Didier Trono, EPFL, Switzerland	RRID:Addgene_12253
Plasmid: pMD2.G	Didier Trono, EPFL, Switzerland	RRID:Addgene_12259
Plasmid: pELNS-CD19.28ζ CAR	This paper	N/A
Plasmid: pELNS-CD19.BBζ CAR	This paper	N/A
Plasmid: pELNS-CD22.28ζ CAR	This paper	N/A
Plasmid: pELNS-CD22.BBζ CAR	This paper	N/A
Plasmid: pELNS-CD33.28ζ CAR	This paper	N/A
Plasmid: pELNS-CD33.BBζ CAR	This paper	N/A
Plasmid: pELNS-CD19.BBζ-FurinP2A-SLC1A5/ASCT2 CAR	This paper	N/A
Plasmid: pELNS-CD19.BBζ-FurinP2A-SLC7A1/CAT1 CAR	This paper	N/A
Plasmid: pELNS-CD19.BBζ-FurinP2A-SLC38A9 CAR	This paper	N/A
Plasmid: PGK-shSLC2A1/GLUT1_EGFP	IGMM, France	N/A
Plasmid: PGK-shSLC1A5/ASCT2_EGFP	IGMM, France	N/A
Plasmid: PGK-shSLC7A1/CAT1_EGFP	IGMM, France	N/A
Plasmid: PGK-shControl_EGFP	IGMM, France	N/A
Software and algorithms		
FlowJo Software 10.8.1	BD Biosciences	N/A
GraphPadPrism 9.4.1	GraphPad Software, Inc.	N/A
Wave	Agilent Technologies	N/A
Image Lab	Bio-Rad	N/A
plateypus	Github	N/A
radianceQuantifier	Github	N/A
PARC (Phenotyping by Accelerated Refined Community-partitioning)	Github	N/A
SINCLAIR: scRNA-seq analysis workflow	CCR Collaborative Bioinformatics Resource (CCBR), NIH	https://github.com/CCBR/SINCLAIR
Deposited Data		
Post-infusion CD19 CAR T-cell post-infusion scRNA-seq dataset	Haradhvala et al., *Nat Med*, 2022^35^	GEO: GSE197268
